# Upgrade of the KWS-2 high-intensity/extended-*Q*-range SANS diffractometer of JCNS for soft matter and biophysics: *in situ* SEC, controlled *in situ* RH/T variation and WANS detection

**DOI:** 10.1107/S160057672500158X

**Published:** 2025-03-19

**Authors:** Jia-Jhen Kang, Ralf Biehl, Georg Brandl, Helmut Korb, Kimio Yoshimura, Vladimir Ossovyi, Andreas Nebel, Jacqueline Lippertz, Ralf Engels, Günter Kemmerling, Alexander Zaft, Hiroki Iwase, Hiroshi Arima-Osonoi, Shin-ichi Takata, Alexander Weber, Simon Staringer, Baohu Wu, Yue Zhao, Stefan Mattauch, Aurel Radulescu

**Affiliations:** a Jülich Centre for Neutron Science (JCNS) at Heinz Maier-Leibnitz Zentrum (MLZ) Forschungszentrum Jülich GmbH Garching 85748 Germany; b Jülich Centre for Neutron Science (JCNS-1) and Institute of Biological Information Processing (IBI-8) Forschungszentrum Jülich GmbH Jülich 52425 Germany; c Department of Advanced Functional Material Research, Takasaki Institute for Advanced Quantum Science, National Institutes for Quantum Science and Technology (QST), Takasaki, 370-1292, Japan; d Peter Grünberg Institute (PGI) and Jülich Centre for Neutron Science (JCNS) Forschungszentrum Jülich GmbH Jülich 52425 Germany; e Jülich Centre for Neutron Science (JCNS-2) Forschungszentrum Jülich GmbH Jülich 52425 Germany; f Neutron Science and Technology Centre Comprehensive Research Organization for Science and Society CROSS Tokai 319-1106 Japan; g Institute for Integrated Radiation and Nuclear Science Kyoto University 2 Osaka 590-0494 Japan; h Materials and Life Science Division Japan Proton Accelerator Research Complex J-PARC Tōkai 319-1195 Japan; Australian Centre for Neutron Scattering, ANSTO, Australia

**Keywords:** SANS, small-angle neutron scattering, WANS, wide-angle neutron scattering, size-exclusion chromatography, SEC–SANS, semi-crystalline polymers

## Abstract

The KWS-2 small-angle neutron scattering (SANS) diffractometer operated by Jülich Centre for Neutron Science (JCNS) at Heinz Maier-Leibnitz Zentrum, Garching, Germany, is dedicated to the investigation of mesoscopic multi-scale structures and structural changes due to rapid kinetic processes in soft condensed matter and biophysical systems. Following requests from the user community, it has been repeatedly upgraded with respect to the most important methodological parameters of an instrument of this type, namely the intensity on the sample, the instrumental resolution and the minimum scattering variable *Q*_min_. Here we report on further specific improvements to the sample environment and detection capabilities which have just been completed or are being implemented. Complementary size-exclusion chromatography (SEC) for *in situ* protein purification was developed and optimized at KWS-2 to provide the instrument with biological samples of controlled quality. The instrument is also currently being equipped with a wide-angle neutron scattering (WANS) detector which will allow it to bridge the atomic and mesoscale, benefiting from the instrument’s adjustable resolution down to Δλ/λ = 2%. For controlled relative humidity and temperature (RH/T) on ionic conductive samples for energy applications or biomembranes for biophysical and health applications, a precise dew point generator has recently been characterized and commissioned; this offers great versatility with regard to varying the contrast *in situ* in the beam on hydrated samples.

## Abstract 2

Upgrades at the KWS-2 small-angle neutron scattering (SANS) diffractometer at Jülich Centre for Neutron Science (JCNS) for an optimal sample quality for biological systems [in-beam size-exclusion chromatography (SEC) complementarity], controlled humidity and temperature (RH/T) on ionic conductive samples and biological membranes, and diffraction capability (wide-angle neutron scattering, WANS) are reported.

## 1. Introduction

The small-angle neutron diffractometer KWS-2 (Radulescu *et al.*, 2012*a* ▸) operated by the Jülich Centre for Neutron Science (JCNS) at the Heinz Maier-Leibnitz Zentrum (MLZ), Garching, Germany, is a high-throughput instrument dedicated to the investigation of mesoscopic multi-scale structures and structural changes in soft condensed matter and biophysical systems. The instrument currently enables a broad *Q* range to be explored, between 2.0 × 10^−4^ and 1.0 Å^−1^, and offers high neutron intensities with MHz detection capabilities (Houston *et al.*, 2018 ▸) and adjustable experimental resolutions (Radulescu *et al.*, 2015*a* ▸) in continuous or time-of-flight (TOF) mode based on the instrument’s optimized neutron guide system (Radulescu *et al.*, 2012*b* ▸), versatile velocity selector, fast detection electronics for the main ^3^He detector and main double-disc chopper. A secondary single-disc chopper (Balacescu *et al.*, 2021 ▸) enables background reduction by facilitating the discarding of inelastically scattered neutrons from hydrogenated samples. The wide *Q* range is covered by the combination of the pinhole mode, with variable wavelength λ between 2.8 and 20 Å and a detection distance *L*_D_ between 1.5 and 20 m after the sample, and the focusing mode, with MgF_2_ parabolic lenses and a secondary high-resolution detector (Radulescu *et al.*, 2012*a* ▸).

In this article, we report recently accomplished and ongoing upgrades to the sample environment and detection capabilities at KWS-2. These include the following: (i) A newly installed size-exclusion chromatography (SEC) complementarity allows for setting a desired sample quality in the beam while working with biophysical systems. This enables in-beam selection of the right species of biological molecules for investigation with neutrons while separating the undesired ones such as aggregates or impurities before they reach the neutron sample cuvette. (ii) To bridge atomic and mesoscale structures, diffraction capabilities are being added for a complete analysis of systems with multiple structural levels such as semi-crystalline polymers. An extended length scale from ångstroms to micrometres will be covered at the same instrument while using the same sample and sample environment equipment. (iii) A new versatile humidity generator was recently acquired, calibrated and implemented in the suite of dedicated ancillaries at KWS-2. This provides the instrument with suitable sample environment conditions to allow significant progress in the field of proton and anion exchange membranes, contributing to the replacement of haza­rdous fluorine-containing membranes such as Nafion with environmentally friendly and safe hydro­carbon membranes. These innovations will enhance the performance of the instrument by overcoming the current lack of specialized ancillary equipment and capabilities for the thorough structural characterization of systems of interest for applications in energy, health and smart materials. They will be available for the user program at KWS-2 when the MLZ FRM II reactor is put back into operation, planned for the end of 2025.

## 2. Instrument description – current performance

**Figure 1:**
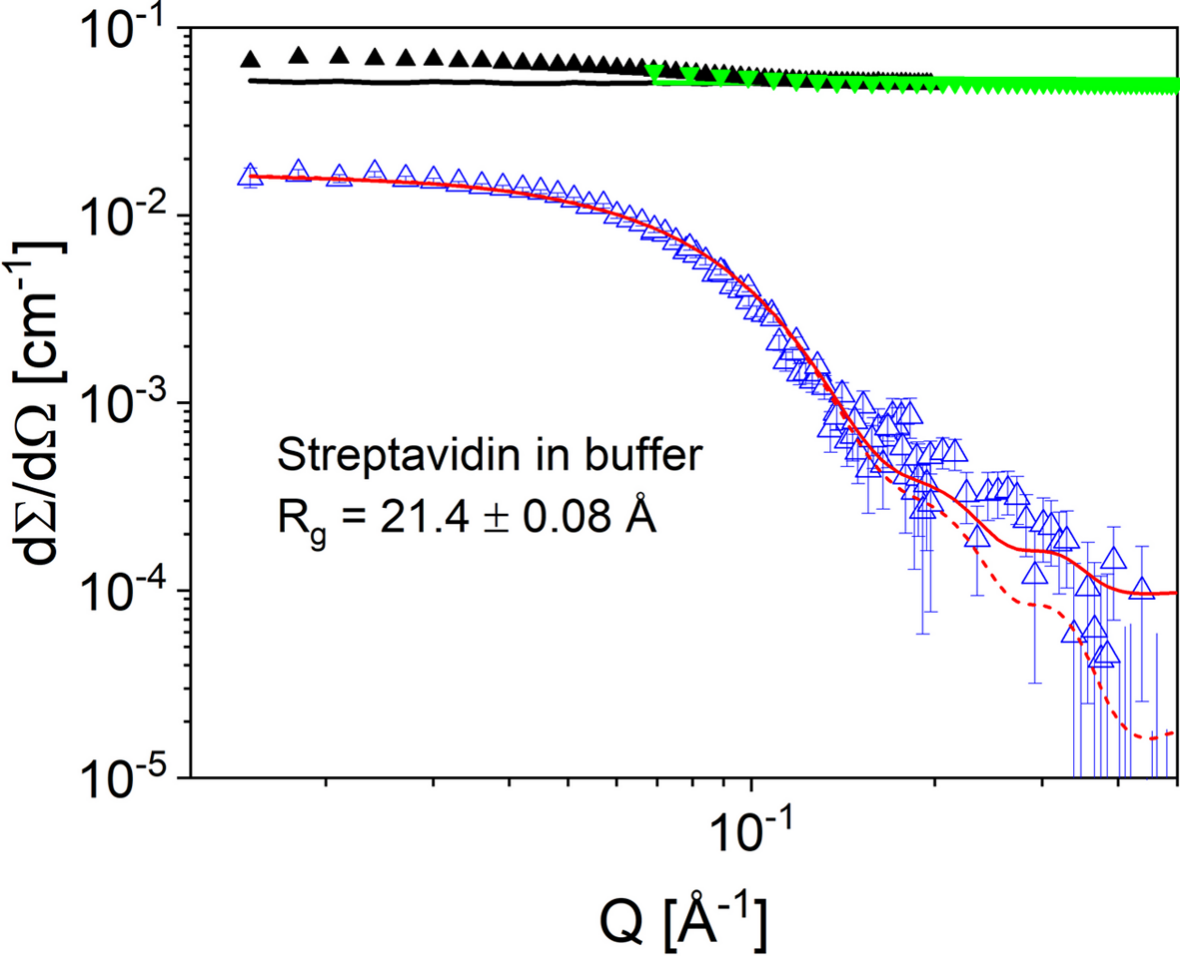
SANS pattern of streptavidin protein (0.5 mg ml^−1^) in buffer solution (Houston *et al.*, 2018 ▸). The green and black solid symbols and line represent the experimental scattering patterns of the protein solution and buffer, respectively, recorded at detection distances of *L*_D_ = 4 m (black) and *L*_D_ = 1.5 m (green) with λ = 5 Å over an acquisition time of 1800 s in each configuration. The open blue symbols show the protein scattering pattern after correction of the buffer contribution. The red curves show the calculated scattering pattern based on the respective PDB structure (PDB code 1n7y; Le Trong *et al.*, 2003 ▸) using the computational code *PEPSI-SANS*, ILL Grenoble (Grudinin *et al.*, 2017 ▸): with (solid) and without (dashed) consideration of the incoherent background.

KWS-2 is supplied with neutrons via a neutron guide that has been optimized to transport high neutron intensities to the sample position. A maximum neutron flux of 2 × 10^8^ neutrons cm^−2^ s^−1^ is available on the sample for λ = 4.5 Å with Δλ/λ = 20% when the shortest collimation length of *L*_C_ = 2 m is used (Radulescu *et al.*, 2012*a* ▸; Houston *et al.*, 2018 ▸). In combination with the fast readout electronics of the ^3^He tube main detector with count rates in the MHz range, this enables the measurement of high-quality scattering data from weak scattering samples such as diluted solutions of small proteins (Fig. 1 ▸).

**Figure 2:**
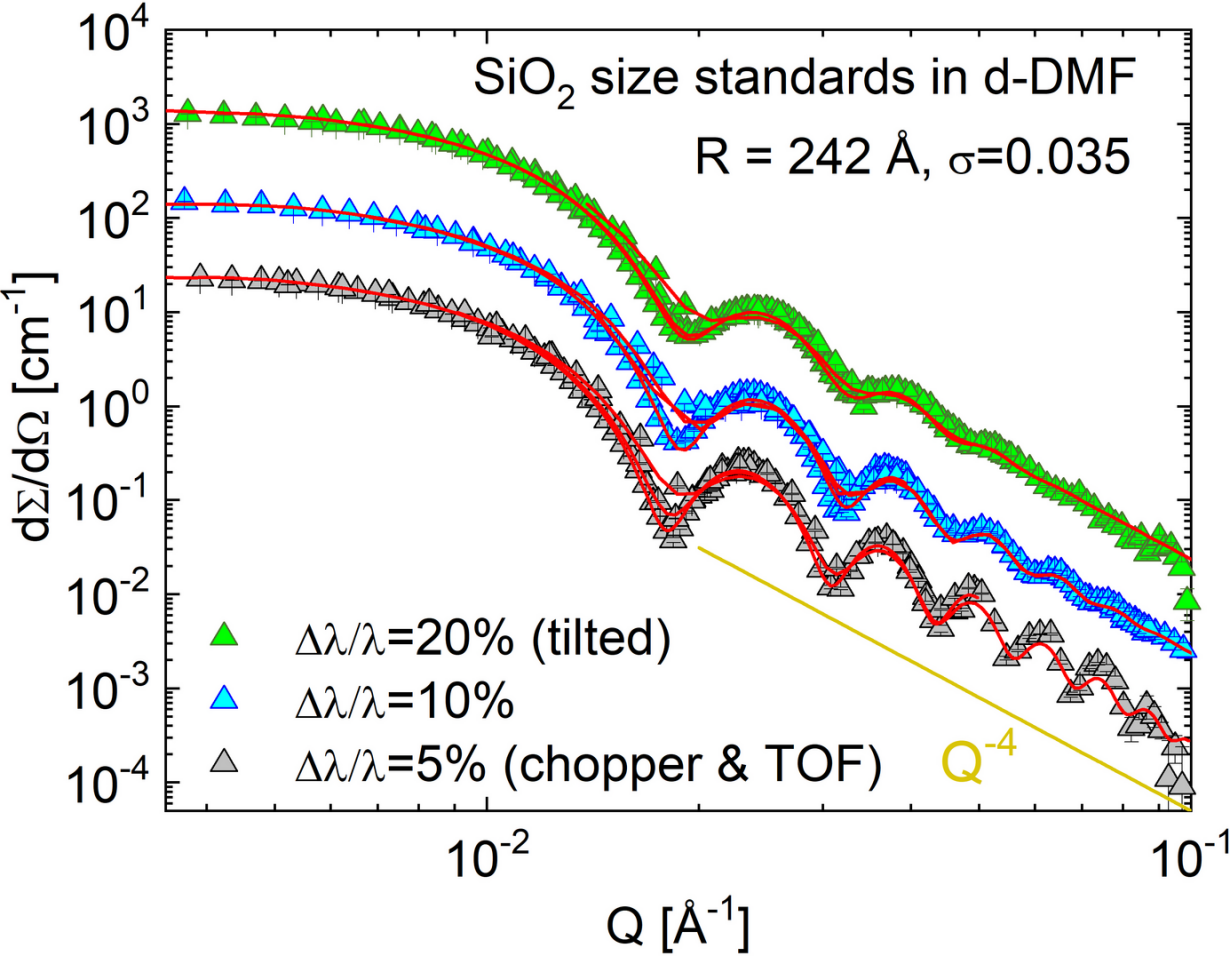
SANS pattern of spherical SiO_2_ particles with low polydispersity σ in deuterated di­methyl­formamide (d-DMF). The experimental data (symbols) were measured at different resolutions as defined by tuning Δλ/λ with the velocity selector in parallel or tilted orientation to the beam axis or the velocity selector chopper tandem at KWS-2. The red curves represent the fit according to equation (9) of Radulescu *et al.* (2015*a* ▸).

The versatile velocity selector (Airbus, Germany) enables an easy selection of the wavelength λ and wavelength spread Δλ/λ, depending on whether the specific scientific experiment demands either an improved resolution, thus Δλ/λ = 10%, or a high intensity, hence Δλ/λ = 20%, by operating it either in the standard configuration, parallel to the beam axis, or in a tilted position with ξ_i_ = −10° with respect to the beam axis. The resolution can be further improved to Δλ/λ = 2%, in a range where no velocity selector can compete (Radulescu *et al.*, 2015*a* ▸; Radulescu, 2024 ▸), by using the TOF mode with the versatile double-disc chopper providing a variable slit opening and a variable frequency, to match the optimal TOF conditions depending on the wavelength λ and the sample-to-detector distance *L*_D_ used. The instrumental resolution at KWS-2 is tunable in a quick and user-friendly manner from the instrument control script; an example is shown in Fig. 2 ▸, where the scattering features of the measured spherical form factor of SiO_2_ size-standard spheres with low polydispersiy in size (*R* = 242 Å, σ = 0.035) are revealed in more detail with improvement of the Δλ/λ between 20% and 5%.

**Figure 3:**
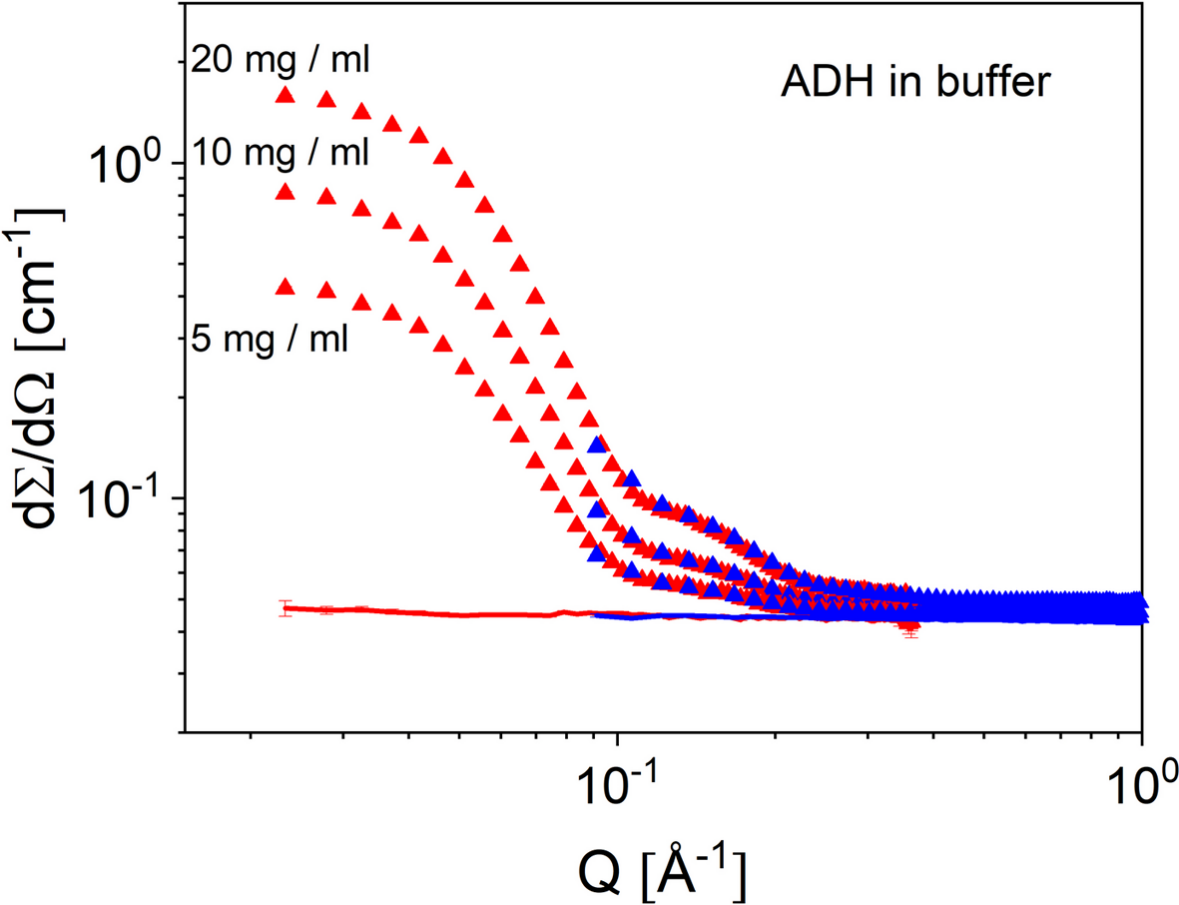
Measured SANS patterns of ADH protein at different concentrations in buffer solution. The symbols indicate the results from the protein solutions, while the lines show the contribution of the buffer solution to the scattering. Results collected at *L*_D_ = 4 m and 1.5 m are shown in red and blue, respectively.

**Figure 4:**
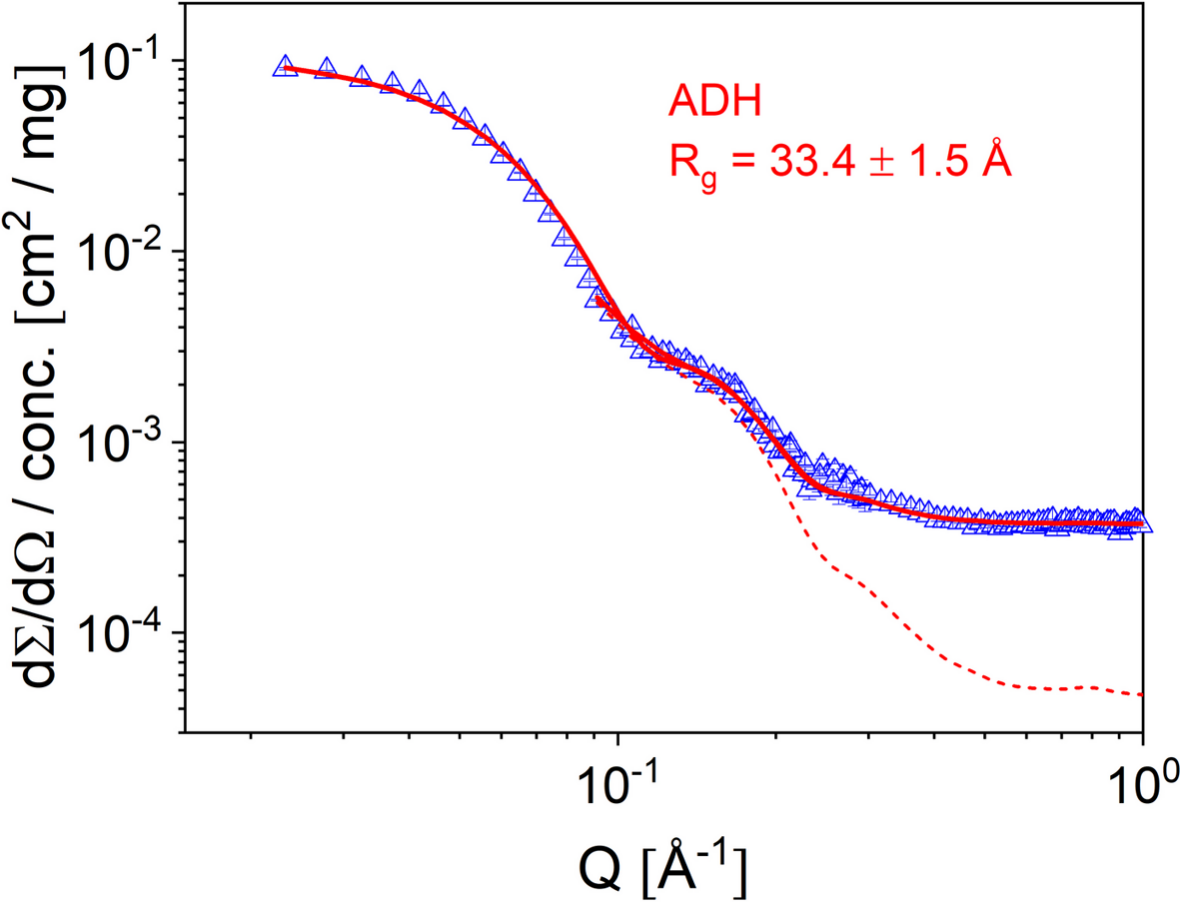
SANS pattern of the ADH protein after correction for the buffer contribution (open symbols) and the scattering patterns calculated with the calculation code *JScatter* (Biehl, 2019 ▸): with (solid) and without (dashed) consideration of the incoherent background.

High-resolution small-angle neutron scattering (SANS) using the TOF mode at KWS-2 is helping in the characterization of long-range-ordered systems such as micellar lattices (Amann *et al.*, 2015 ▸), of interest in gel-type pharmaceutical formulations (Puig-Rigall *et al.*, 2020 ▸; Puig-Rigall *et al.*, 2021 ▸). Tilting of the velocity selector with respect to the beam axis shifts the λ_min_ available at the instrument from 4.5 Å in the standard configuration to 2.8 Å, which enables a *Q*_max_ = 1.0 Å^−1^ to be currently reached. This is beneficial for the study of small morphologies such as nanoscale water clusters in various matrices (Schiavone *et al.*, 2023 ▸; Pabst *et al.*, 2021 ▸). For proteins in solution this is particularly interesting, because a detailed analysis can be carried out in terms of structural models while considering their coherent and incoherent scattering contributions after corrections for the instrumental and buffer contributions have been applied. Figs. 3 ▸–4 ▸ show the scattering from alcohol de­hydrogenase (ADH) protein (Sigma–Aldrich) in buffer solution at different protein concentrations (5, 10 and 20 mg ml^−1^; Fig. 3 ▸) and the protein scattering after correction for the buffer contribution was applied, together with the coherent and total scattering contributions, as calculated from the atomic Protein Data Bank structure (PDB entry 4w6z; Raj *et al.*, 2014 ▸) up to *Q* = 1.0 Å^−1^ (Fig. 4 ▸).

After scaling by concentration, the buffer-corrected data overlap except at low *Q* where a small influence of the structure factor was observed in the case of the higher concentrations. For modeling of the form factor we considered the lowest concentration for the low-*Q* region, to reduce structure-factor effects, and the largest concentration for high *Q* because of the improved statistics.

For the form factor calculation based on the respective PDB structures, the computational code *Jscatter* (Biehl, 2019 ▸) was used. The data were smeared according to Pedersen *et al.* (1990 ▸) taking into account the wavelength spread Δλ/λ = 14% (Radulescu, 2024 ▸) and the geometry of the instrument for the two selected detector distances. A good aggrement between the PDB structure and measured data was found with regard to main scattering features (Fig. 4 ▸): the Guinier regime at low *Q*, the characteristic two shoulders at 0.15 and 0.28 Å^−1^, and the plateau at higher *Q* which is strongly dominated by the incoherent contribution. The code calculates the incoherent scattering based on all atoms present in the atomic structure, including the incoherent contribution from a larger density in the hydration layer.

Finally, a *Q*_min_ = 2 × 10^−4^ Å^−1^ is enabled by the focusing mode at KWS-2. The parabolic MgF_2_ lenses placed 1.5 m in front of the sample are used at 70 K to improve transmission by suppressing the scattering of phonons in the lens material. These are combined with a small entrance aperture (2 × 2 mm) at *L*_C_ = 20 m and a high-resolution scintillation detector (HRD) at *L*_D_ = 17 m, with a position resolution of 1 mm. The HRD is parked in a tower of the detector tank (Radulescu *et al.*, 2012*a* ▸) and can be brought into the beam on demand when the main SANS detector is parked at 20 m. The thorough characterization of polymer gels (Kaneko *et al.*, 2024 ▸; Talley *et al.*, 2019 ▸) or of complex assemblies of proteins with polymers or lipids (Krugmann *et al.*, 2020 ▸) over a wide size range benefits from the advantages of combined focusing and pinhole modes on the same instrument. This is particularly important for the structural examination of samples that have undergone very specific thermal or compositional preparation, for which a high degree of data reproducibility would not be achieved if an additional beamline were to be included in the study to cover a wide *Q* range.

## 3. New upgrades

### 3.1. In-beam SEC complementarity

For systems that dynamically form into oligomer states or systems that are prone to forming aggregates, the collected small-angle scattering (SAS) data contain scattering from both the aggregates and the individual molecules. For such systems, an *in situ* fractionation method is necessary before the SAS measurement, in order to separately collect scattering data of different species. SEC is a technique to fractionate particles by size in a mixture. A SEC instrument is typically equipped with a pump which provides a steady flowrate of the elution buffer, pushing the injected sample solution through the elution column. The column is stuffed with gel particles with voids having specified sizes. During the elution of the sample, large particles are flushed out of the column at an early flushing time because they are excluded from the small voids, while small particles are flushed out of the column at a late flushing time due to the relatively long traveling path, as they travel through the small voids in the column. Downstream of the column, a UV–vis detector records the flushing time of different-sized particles, which is used to examine the fractionation performance of the SEC instrument.

*In situ* SEC combined with SAS has been achieved, with continuous upgrades, at synchrotron X-ray facilities worldwide (Ryan *et al.*, 2018 ▸; Thureau *et al.*, 2021 ▸; Shih *et al.*, 2022 ▸); in addition, there are several successful setups with in-house small-angle X-ray scattering (SAXS) machines (Bucciarelli *et al.*, 2018 ▸; Inoue *et al.*, 2019 ▸). The SEC–SAXS technique has made a large contribution to the biomedical field because of its ability to resolve structures of individual biomolecules. For SANS, the first ever SEC–SANS setup worldwide was achieved at the D22 instrument at the Institut Laue–Langevin (ILL) (Jordan *et al.*, 2016 ▸; Martel *et al.*, 2023 ▸). Compared with SEC–SAXS, the benefit of SEC–SANS is the opportunity to perform contrast matching and deuterium labeling for the targeted molecules, and this is especially suitable for resolving the structure of multi-component biomolecules (Johansen *et al.*, 2018 ▸).

**Figure 5:**
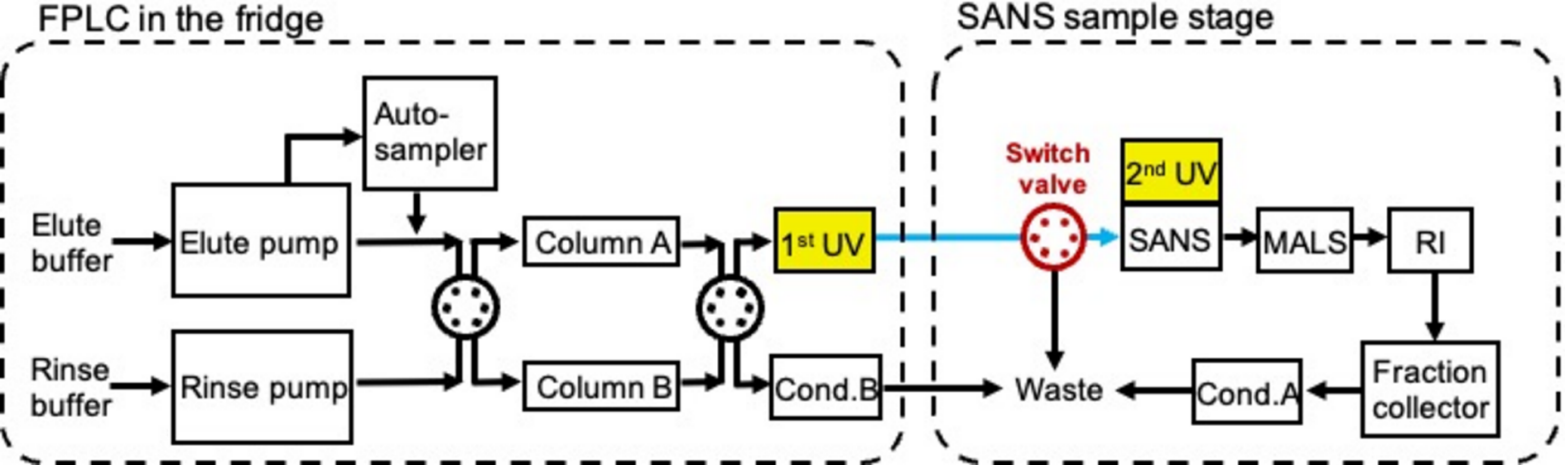
SEC–SANS flowchart at KWS-2, showing the sample flow path and the key components. The yellow blocks mark the two UV detectors at the FPLC and at the SANS stage. The blue line indicates the connecting capillary transferring the sample from the FPLC system to the SANS measurement. The switch valve upstream of SANS is marked in red.

At KWS-2 a SEC instrument has now been established in-line with SANS, which will provide a SEC–SANS combination to the user community when the FRM II reactor resumes its activity. The design of the SEC–SANS instrumental setup is shown in the flowchart in Fig. 5 ▸. The chromatography is performed with the Knauer AZURA fast protein liquid chromatography (FPLC) system, which is equipped with an auto-sampler, a binary pump as the elution pump, an isocratic pump as the rinse pump, a UV–vis detector, a refractive index (RI) detector, two conductivity detectors and a fraction collector. The setup is furthermore combined with a multi-angle light scattering (MALS) machine. The UV–vis detector, referred to as ‘1st UV’ in the following, is connected downstream of the elution column, with a light path of 3 mm. The column used for all the test measurements was a Superdex200 10/300 (Cytiva, L × i.d. 30 cm × 10 mm), suitable for protein molar mass within the range of 10–600 kDa. Due to the limited space around the sample stage of KWS-2, the FPLC system, equipped with the 1st UV detector [Fig. S1(*a*) in the supporting information], is located on an elevated platform above the experiment hutch [Fig. S1(*b*)], with a distance of 2.5 m to the SANS sample cuvette. A switch valve (Fig. 5 ▸, red valve) is installed between the 1st UV and the SANS flow cuvette, which allows only the fractions of interest to flow into the SANS flow cell for neutron exposure and SANS data collection. This way, contamination of the cell by the unwanted fractions is avoided, and thus the SANS data of the molecules of interest are not compromised.

The SANS cuvette holder is mounted on the sample stage [Fig. S1(*c*)], where a second UV–vis spectrometer, the ‘2nd UV’, is installed to monitor the samples flowing through the SANS sample cuvette. It consists of a light source (DH-2000-DUV lamp, 190–2500 nm, Ocean Optics), a spectrometer (FX UV–Vis, Ocean Optics), and optical fibers and collimating lenses (74-UV, Ocean Optics). The four-sided polished SANS flow cuvette is placed inside a thermostated holder with Peltier elements, where the solution capillaries and the cuvette are sealed together using ep­oxy resin AB glue. The design of the SEC complementarity at KWS-2 follows the boundary conditions imposed at the sample position of the instrument. The main components and processes were optimized for providing the SANS instrument with the desired sample quality, also providing the users with information on the realistic initial conditions required by the sample purification process. An obvious drawback of the setup at KWS-2 compared with similar setups at other beamlines is the large dead volume of the system. Dead volume is the total volume through which the sample flows in the system, and it is strongly related to the separation performance of FPLC. When the sample solution is injected into the FPLC instrument and flows in the capillary, it is concentrated; therefore a sharp UV absorbance peak is detected by the 1st UV. After flowing through a long distance in the SANS cuvette, it becomes diluted by the continuous flow with the elution buffer, resulting in a broadening of the elution peak. The longer the traveling distance for the injected sample, *i.e.* the larger the dead volume, the more diluted the sample and the more serious the peak broadening effect. Thus, to improve the separation efficiency of FPLC instruments, the dead volume of the system was minimized following detailed test measurements. The fractionation performance of the FPLC system is often evaluated by the dilution factor of the injected sample solution, *i.e.* the original concentration divided by the concentration after elution.

**Figure 6:**
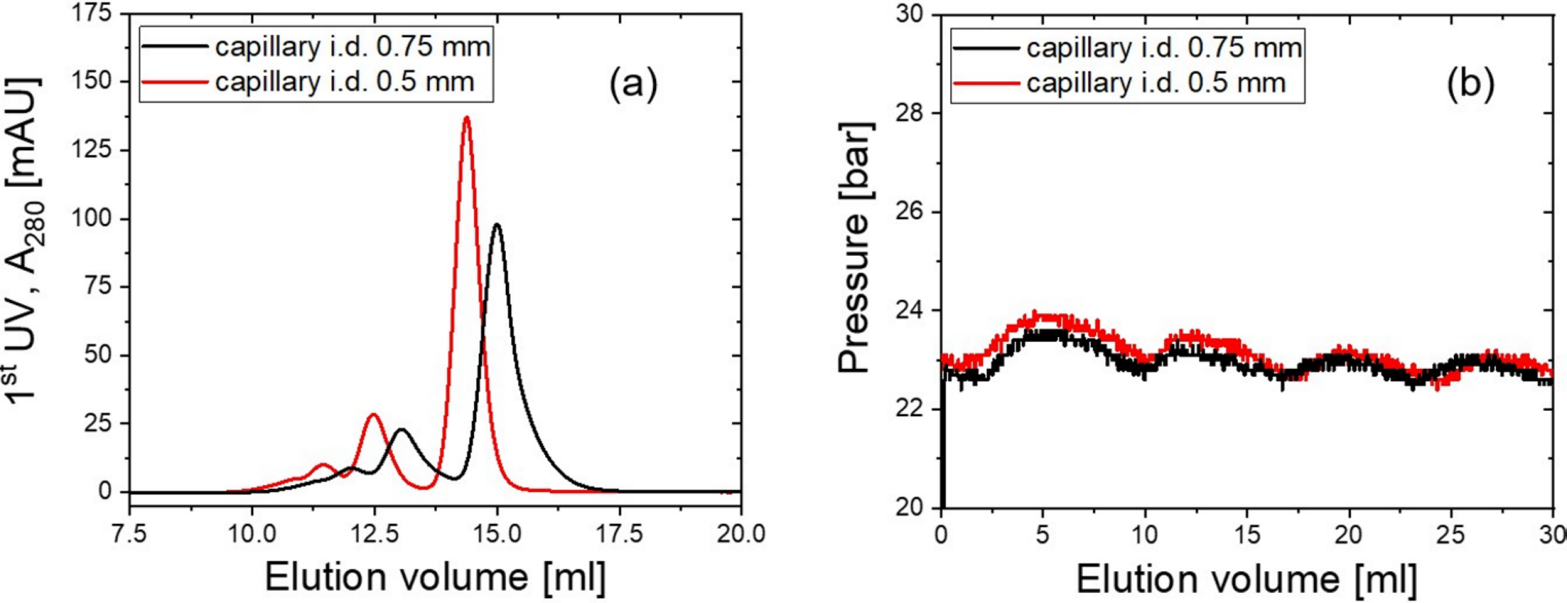
(*a*) Elution profiles of 10 mg ml^−1^ BSA solution with capillary i.d. = 0.75 mm (black curve) and i.d. = 0.5 mm (red curve). (*b*) The corresponding pump back pressure during the measurement.

The FPLC system at KWS-2 functions with its solution capillary having an inner diameter (i.d.) of 0.75 mm. A test of its fractionation efficiency was performed with a 10 mg ml^−1^ bovine serum albumin (BSA) solution and an injection volume of 200 µl, eluted by phosphate buffered saline (PBS). The elution profile [Fig. 6 ▸(*a*), black curve], expressed by plotting UV absorbance at 280 nm, *A*_280_, along the elution volume, shows the signal of large aggregates and peaks of BSA trimer, dimer and monomer, from low to high elution volume. The asymmetric monomer peak and the poor separation between peaks are probably due to the peak broadening effect resulting from the large dead volume of the system. The monomer concentration at its peak position is thus calculated to be 1.52 mg ml^−1^.

For a better chromatography performance, a smaller dead volume of the system was achieved by replacing the capillaries with ones having an i.d. of 0.5 mm. The elution profiles after changing the capillaries [Fig. 6 ▸(*a*), red curve] show symmetric elution peaks and improved peak separation. The monomer peak gives a concentration of 2.12 mg ml^−1^, *i.e.* an increase in concentration of 40% compared with before the capillary replacement. However, reducing capillary i.d. comes with the risk of an increase in the pump back pressure, which might put the column in danger. The back pressure change before and after replacement of the capillaries was inspected. It was observed that although the back pressure, measured at the elution pump, with i.d. = 0.5 mm [Fig. 6 ▸(*b*), red curve] is slightly larger than that with i.d. = 0.75 mm [Fig. 6 ▸(*b*), black curve], it stays well below the bearing limit of the column, *i.e.* 50 bar. Considering the dilution factor of the BSA monomer, the monomer concentration in the sample is obtained from the integration fraction of the monomer contribution in the elution profile. For the measurement with capillary i.d. = 0.75 mm, the monomer concentration in the injected sample solution is 7.35 mg ml^−1^. Assuming the monomer concentration after elution to be 1.52 mg ml^−1^, the dilution factor was calculated to be 4.84. For the measurement with capillary i.d. = 0.5 mm, the monomer concentration in the injected sample solution is 7.37 mg ml^−1^, and the monomer concentration after elution is increased to 2.12 mg ml^−1^. The change leads to a reduced dilution factor of 3.48.

The monitoring of the arrival of the fractionated sample from the FPLC system to the SANS sample cuvette is achieved with the 2nd UV which provides *in situ* information about the sample in the flow cuvette during the SEC–SANS measurement. Considering the spatial separation between the FPLC instrument and the SANS stage, it is important to check how much the UV signal changes from the 1st to the 2nd UV after the sample flows through the 2.5 m capillary (i.d. = 0.25 mm).

**Figure 7:**
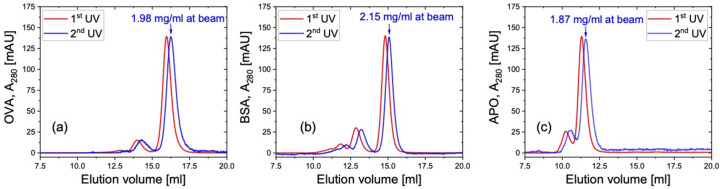
Elution profiles of 10 mg ml^−1^ (*a*) OVA, (*b*) BSA and (*c*) APO. The maximum monomer concentration from the 2nd UV profile is given above the corresponding elution peak. All *A*_280_ are normalized by the light path.

Measurements of the elution profiles were carried out on different protein solutions with the 2nd UV aiming at the front face of the SANS flow cuvette to learn about the protein concentration for SANS data collection. A customized flow cuvette with a light path of 1 mm for the neutron beam and a small inner volume of around 135 µl was used for the SEC–SANS measurements [Fig. S1(*c*)]. Three proteins with different molar mass were chosen for the measurements: chicken ovalbumin, OVA (45 kDa), BSA (66 kDa) and apoferritin, APO (454 kDa). The elution profiles of the 10 mg ml^−1^ solutions from the 1st UV and the 2nd UV are shown in Figs. 7 ▸(*a*), 7 ▸(*b*) and 7 ▸(*c*), respectively. The UV absorbance at 280 nm, *A*_280_, is normalized by the light path of the two devices for comparison. Note that, for all three samples, there are only small changes between the 1st UV and the 2nd UV. Thus, despite the long capillary between the FPLC instrument and the SANS sample stage, the separation resolution of the chromatography does not worsen throughout the flow. For each sample, the maximum *A*_280_ of the monomer peak from the 2nd UV profile is used to calculate the monomer concentration, so that the protein concentration at the neutron beam can be estimated from
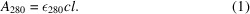
Here *c* is the concentration, *l* is the light path, and the extinction coefficients at 280 nm ε_280_ for 0.1% solution and 10 mm light path are ε_0.1%, OVA_ = 0.701 (Pace *et al.*, 1995 ▸), ε_0.1%, BSA_ = 0.646 and ε_0.1%, APO_ = 0.729 (Graewert *et al.*, 2020 ▸) for the three proteins. The calculation gives 1.98 mg ml^−1^ for the OVA monomer peak, 2.15 mg ml^−1^ for the BSA monomer peak and 1.87 mg ml^−1^ for the APO monomer peak. Dividing the monomer concentration in the original sample by the monomer concentration obtained at the 2nd UV, the dilution factor for OVA, BSA and APO is found to be 4.37, 3.40 and 4.53, respectively; these vales are comparable to those of other SEC–SAS instruments. The small UV absorbance variation from the 1st UV to the 2nd UV is attributed to the thin i.d. (0.25 mm) of the connecting capillary as well as the small inner volume of the flow cell, which together help to suppress the dilution effect. The presented experimental results provide information on the sample condition for SEC–SANS measurements at KWS-2: with an injected protein concentration of 10 mg ml^−1^ and volume of 200 µl, the obtained concentration in the center of the flow cell for neutron exposure will be around 2 mg ml^−1^, with a dilution factor around 4; note also that the actual experimental results may vary depending on the individual samples.

**Figure 8:**
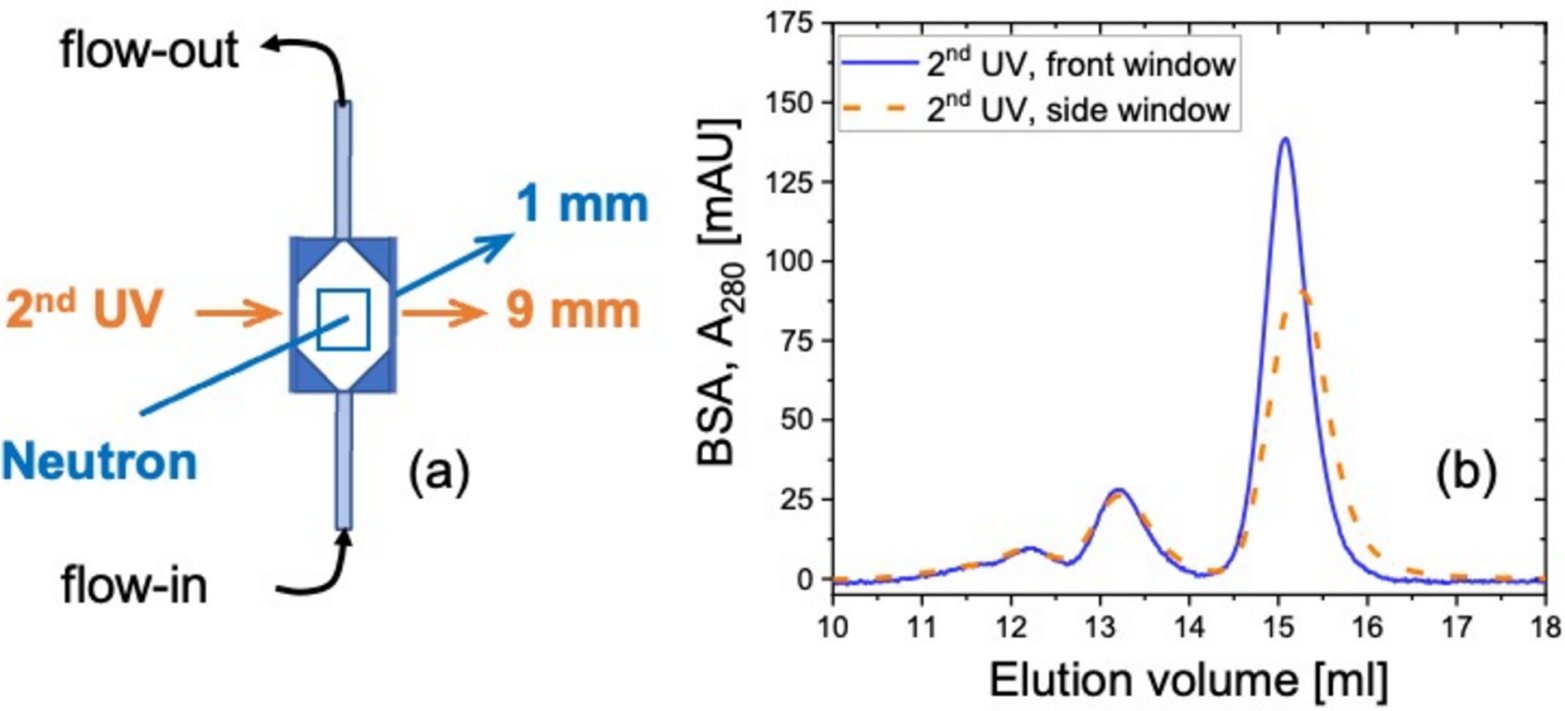
(*a*) Schematic demonstration of how the neutron beam and the 2nd UV light penetrate the flow cell during practical SEC–SANS measurements. The corresponding light path is given at the side. (*b*) Elution profiles of 10 mg ml^−1^ BSA solutions collected from the 2nd UV when light penetrates the SANS flow cell on the side window (dashed orange curve) and on the front window (solid blue curve) are plotted for comparison. All *A*_280_ are normalized by the light path.

During practical SEC–SANS measurements for commissioning, the 2nd UV will be installed in such a way that the light penetrates the flow cell on the direction perpendicular to the neutron beam [schematically demonstrated in Fig. 8 ▸(*a*)]. This means it goes through the side window of the cell, where the light path is 9 mm. To inspect the feasibility of such a configuration, the experiments mentioned above were repeated, with the UV light penetrating the side window of the flow cell. Data collected by the 2nd UV at the side window [Fig. 8 ▸(*b*), dashed orange curve] are plotted together with those collected at the center of the front window [Fig. 8 ▸(*b*), solid blue curve], and *A*_280_ is normalized by the light path. The distinct elution peaks collected at the side of the flow cell align well with those collected at the front window, suggesting that the signal collected from the 2nd UV serves as a good indicator for *in situ* monitoring with simultaneous SANS measurements. *A*_280_ of the elution peaks is smaller when data are collected at the side of the flow cell, especially for monomers, indicating a dilution effect along the horizontal direction during elution. UV signal at different positions along the vertical direction was also detected (*i.e.* along the sample flow direction), showing a rather small dilution effect (Fig. S2). Considering the typically applied neutron beam of 4 × 6 mm at KWS-2, with a shorter size along the horizonal direction, the aim of obtaining single particle scattering of the targeted protein should be achievable. However, the feasibility measurement will be carried out with neutrons, and the SANS data obtained in this way will be analyzed thereafter.

**Figure 9:**
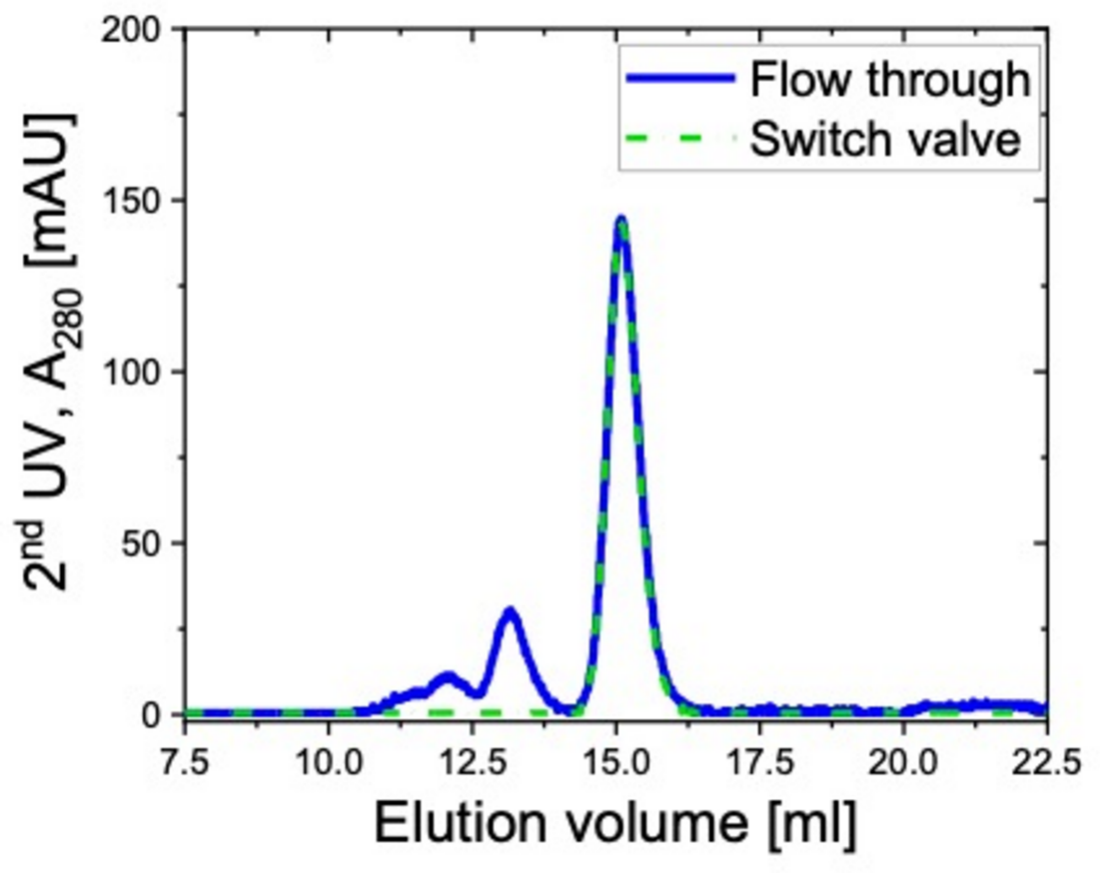
Elution profiles of 10 mg ml^−1^ BSA utilizing the switch valve (dashed green curve) and not utilizing it (solid blue curve). *A*_280_ is collected from the 2nd UV at the front window of the flow cell.

A switch valve installed upstream of the SANS flow cell serves to control the flow path so that only the elution peak of interest flows into the cell for SANS data collection, while the remaining elution peaks are directed to the waste (see Fig. 5 ▸). Elution measurements on 10 mg ml^−1^ BSA solution were performed to demonstrate the concept, where the switching function was programmed in the FPLC controlling software. During the ‘flow through’ measurement, where the switch valve was not utilized and all the species after chromatography go into the flow cell, the elution profile from the 2nd UV shows signal from aggregates, trimers, dimers and monomers (Fig. 9 ▸, solid blue curve). In another measurement where the switch valve was utilized, the valve position shifts according to the elution volume (EV). Between 0 and 8.5 EV, the solution flows into the SANS flow cell, so that SANS data of the buffer flowing through the cell can be collected to facilitate the SANS data reduction. Then, between 8.5 and 14.0 EV, the flow was guided to the waste, as the structural information of the aggregates, trimer and dimers is not of interest in the current case. Finally, between 14.0 and 25 EV, the valve shifts again to send the monomers into the cell for SANS data collection. Thus, the obtained elution profile from the 2nd UV shows a single peak attributed to the monomers (Fig. 9 ▸, dashed green curve). The fact that the two elution profiles overlap nicely in the EV range of the monomer peak indicates that the influence on the sample from the additional valve on the flow path is small and negligible.

With a maximum flux of 2 × 10^8^ neutrons cm^−1^ s^−1^ at the sample position at KWS-2, combined with the rapid readout detection electronics capable of working in list mode, the buffer-subtracted scattering curve of protein solution as dilute as 0.5 mg ml^−1^ (Fig. 1 ▸) shows rather well defined scattering features. On the basis of this result, the SEC–SANS setup at KWS-2 will enable collection of clear scattering curves for samples with a concentration above 1.2–1.5 mg ml^−1^ after chromatography when the user program at MLZ is resumed. Still, the data quality depends on several factors of the sample, *e.g.* scattering contrast and molar mass. A thorough discussion including MALS and RI analysis will be reported elsewhere.

### 3.2. In-beam relative humidity and temperature variation on the sample

The characterization of polymeric films and membranes under controlled humidity conditions has received significant attention, especially in energy-relevant studies (Bose *et al.*, 2011 ▸; Grzybek *et al.*, 2024 ▸; Song *et al.*, 2022 ▸; Adamski *et al.*, 2021 ▸; Luo *et al.*, 2020 ▸).

Proton- and anion-exchange polymeric membranes produced by various methods to target improvement in either the conductivity in various temperature (T) and relative humidity (RH) conditions or durability after exposure to the application conditions have been intensively studied at KWS-2 (Zhao *et al.*, 2021 ▸; Zhao *et al.*, 2022 ▸; Yoshimura *et al.*, 2024 ▸). However, most of the studies were focused on membranes in the saturated state (equilibrated) in H_2_O, D_2_O or mixtures; contrast variation was applied to get a much clearer view of the structure and morphology of the polymer material and water domains in these conditions. Fewer studies have dealt with in-beam variation of RH and T on the sample due to the lack of a robust, competititve and versatile sample environment to provide full control of the variation of RH, T and contrast (type of hydration water) on the sample in-beam, over a long time. The need for such a neutron scattering dedicated sample environment for the microstructural and microdynamical characterization of various materials has increased, particularly with the advent of intensive studies for finding a non-haza­rdous and environmentally friendly alternative to perfluorinated Nafion.

From experience gained at J-PARC, where a D_2_O/H_2_O vapor generator for contrast-variation neutron scattering was implemented with appropriate sample chambers at different instruments, such as SANS, reflectivity and quasi-elastic neutron scattering, we decided to incorporate a similar humidity generator in the user program at KWS-2 for applications on hydrated polymer or biological membranes. The central piece of the setup is a precise dew point generator which was customized for neutron scattering purposes by the company microEquipment (Tokyo, Japan); this will be used in the near future at KWS-2, when the user program at the FRM II reactor is resumed. It is coupled to a multi-position sample chamber which is thermostated itself for providing the required temperature on the samples in the beam and which requests the appropriate dew point conditions at the generator for obtaining the desired RH on the sample [Fig. S3(*a*)]. The experimental setup for SANS studies under controlled RH/T/contrast conditions is described in detail by Arima-Osonoi *et al.* (2023 ▸), including the working principle, calibration and efficiency on some selected scientific cases. We have successfully used the same setup for the thorough structural characterization by SANS of functionalized (proton-conductive) syndiotactic polystyrene membranes under various RH/T/contrast conditions (Schiavone *et al.*, 2023 ▸). The precise dew point generator incorporates two identical vapor-generation systems, each capable of independently producing D_2_O and H_2_O vapor, and comprises four gas lines with mass flow controllers and valves, two saturators, and a proportional–integral–derivative (PID) controller to regulate the gas flow rate and temperature. Vapors with dew points ranging from +5 to +85°C are generated and two distinct techniques – the two-temperature and divided-flow methods – are applied to obtain the required RH on the sample (Arima-Osonoi *et al.*, 2023 ▸).

The generated humidity is transported to the neutron scattering instrument via a 4 m-long supply line, which consists of flexible stainless steel pipes with a diameter of 3/8 inch, and it is insulated with an Aeroflex plate. All moisture gas lines are equipped with silicone rubber heaters that can heat up to 100°C. As in the case of the SEC complement, this long tube overcomes the space limitations at the KWS-2 beamline sample position which prevent placement of the generator near the sample chamber within the shielded hutch. Several components, including the PID controller and valves, are controlled and monitored by a programmable logic controller that interfaces with the KWS-2 instrument control system. This integration facilitates remote data acquisition and the automated running of experiments. According to the experience obtained at J-PARC, the generator features, the storage tanks and the automatic water pumps will allow several days of continuous operation without manual water supply.

**Figure 10:**
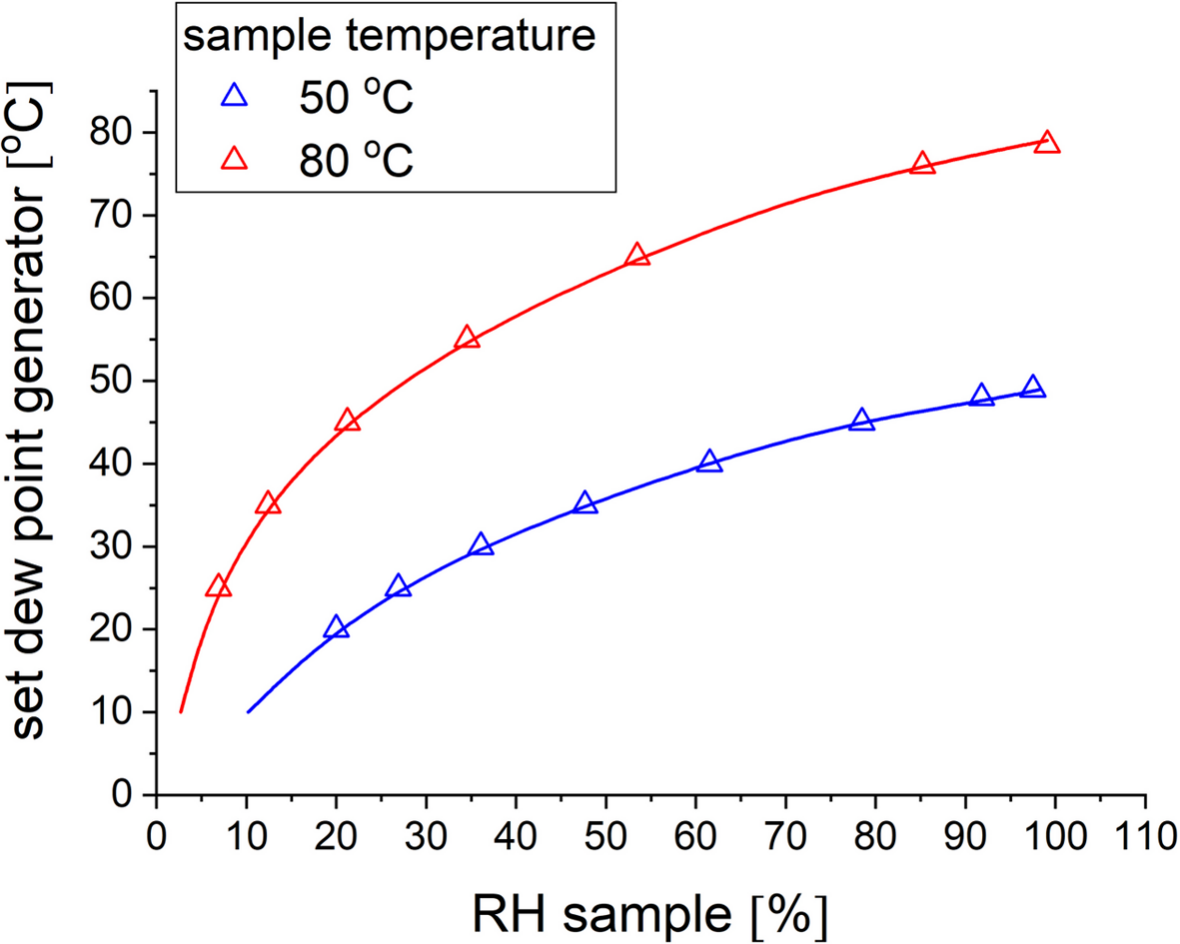
Selected calibration curves of the humidity generator for the sample chamber at different temperatures: the dew point set at the generator versus measured RH in the sample chamber with the dew point mirror sensor for H_2_O vapors (symbols) and D_2_O vapors (lines).

For KWS-2, a multi-position thermostated sample chamber was designed to be used in combination with the humidity generator [Fig. S3(*b*)], allowing the measurement of multiple samples at controlled RH and T conditions. The chamber is equipped with thin Al windows (0.5 mm) and water-circulating internal channels to allow precise temperature control within ±0.5°C between room temperature and 85°C via a Julabo water bath. The system was calibrated with a high-precision Michell S8000 chilled mirror hygrometer (PST GmbH, Friedrichsdorf, Germany) to determine the dew point that must be set on the generator to obtain the desired RH on the sample as a function of sample temperature (Fig. 10 ▸). No observable difference in calibration curves for the H_2_O and D_2_O was obtained. The information from the mirror hygrometer is stored in the recorded data file after the SANS measurement together with the information set on the generator and the sample temperature. Since a detailed characterization of a similar humidity generator performed at J-PARC has already been reported elsewhere (Arima-Osonoi *et al.*, 2023 ▸), we report here the results of a specific experiment to demonstrate the efficiency of the newly achieved humidity generator at KWS-2. This involved measuring the conductivity of Nafion117 membranes at different RH and T conditions using a BT-115 four-electrode cell from Electrochem Inc. coupled to the humidity generator (Fig. S4) and comparing it with known literature data. A Hioki IM3523 LCR meter (Ueda, Japan) was used to interpret the data from the conductivity cell, while a TC-3000 controller from AS ONE (Osaka, Japan) was used to control the temperature on the conductivity cell. The choice of Nafion117 was due to the known proton conductivity of this type of membrane at various RH and T conditions, which enables us to assess the performance of the humidity generator at optimal measurement conditions.

**Figure 11:**
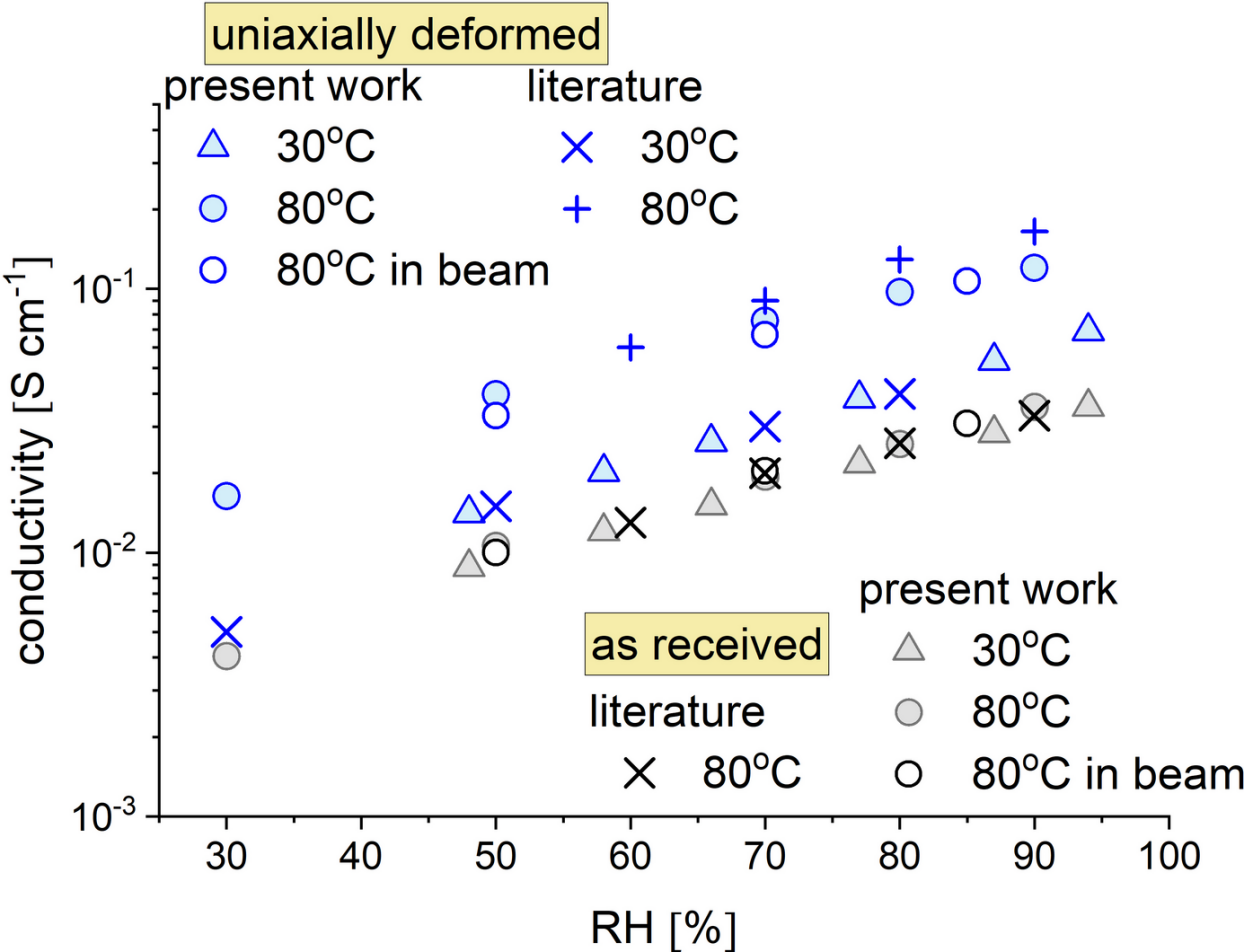
Evolution of ionic conductivity measured on Nafion117 membranes as received (black symbols) and uniaxially deformed (blue symbols), as a function of the RH on the sample (obtained with H_2_O vapors): data measured at 30°C (triangles) and 80°C (circles). The data at 80°C were collected either as part of laboratory measurements at JCNS (full symbols) or in the neutron beam (empty symbols), simultaneously with SANS, at J-PARC. The × and + symbols represent literature data (same color code): black × Wang *et al.* (2002 ▸); blue × Klein *et al.* (2013 ▸); blue + Park *et al.* (2011 ▸). The conductivity measured in-plane along the deformation axis is displayed for the uniaxially deformed membranes.

Fig. 11 ▸ shows the measured proton conductivity at different RH and T values of a Nafion117 membrane, as received (Chemours, Wilmington, DE, USA), parallel to that of a uniaxially deformed Nafion117 membrane (in-plane along the deformation axis). The deformed membrane was prepared with a deformation ratio of 2.0 compared with the initial length using a Linkam Modular Force Stage (Linkam Scientific Instruments, Salfords, UK). Both data sets are compared with literature data on the same systems as reported by Wang *et al.* (2002 ▸) for the Nafion117 membranes and by Park *et al.* (2011 ▸) and Klein *et al.* (2013 ▸) for the stretched Nafion117 membranes.

There is good agreement between our data and the literature data in terms of both the quantitative values and the qualitative RH and T behavior. Our data confirm the known behavior of Nafion117 membranes, which show increasing conductivity values with increasing RH and only a very small difference between the conductivity values at 30 and 80°C, as reported by Sone *et al.* (1996 ▸). Furthermore, the striking difference between the conductivity values at 30°C and those measured at 80°C in-plane along the deformation axis on stretched Nafion117 membranes indicates that our data are consistent with those of Park *et al.* (2011 ▸) and Klein *et al.* (2013 ▸). The much higher values observed at 80°C, which increase with increasing RH, must be related to structural features of such deformed membranes. The water accumulated at high temperature apparently organizes in oriented domains, a morphology that facilitates the development of consistent and continuous conductive paths along the deformation direction of the membrane, as reported by Allahyarov & Taylor (2009 ▸). The good performance of the newly implemented humidity generator for KWS-2 was further confirmed by the conductivity measurement on the same Nafion117 membranes using the similar humidity generator at J-PARC and the in-beam conductivity cell with a Hioki 3532-80 chemical impedance analyzer at the BL-15 Taikan SANS–WANS (wide-angle neutron scattering) beamline (empty circles in Fig. 11 ▸; for the cell setup see Fig. S5). Although the simultaneous SANS and conductivity measurement data from these Nafion samples, including the contrast variations applied to the hydrated domains, will be the subject of a forthcoming article, Fig. S6 shows a selected set of SANS data to demonstrate the strength of the experimental approach we have used. The scattering patterns from the as-prepared sample and the uniaxially deformed sample are similar to those reported by Rubatat & Diat (2007 ▸). The four main scattering features of Nafion membranes for the as-received film sample can be clearly identified from the experimental data: at low* Q*, the power-law characteristic of the large-scale fractal morphology of the membrane (1); at medium *Q*, the matrix knee characteristic of the scattering length density variation between crystalline and amorphous domains in the material (2); at higher *Q*, the ionomer peak indicating the correlation between the ion clusters in the hydrated domains of the membrane (3); and at very high *Q*, the first crystalline peak due to interchain correlations in the crystalline matrix of the material (4). After uniaxial deformation of the membrane, the intensity of the ionomer peak increases along the direction perpendicular to the deformation axis, an effect also observed by Rubatat & Diat (2007 ▸) and explained by the alignment of the scattering objects along the deformation axis and the formation of parallel arrangements. Moreover, the first crystalline peak is prominent on the axis perpendicular to the deformation direction, which has also been observed by Mendil-Jakani *et al.* (2015 ▸). This is related to the orientation of the crystalline lamellae along the deformation direction, such that the main crystalline planes are oriented perpendicular to the deformation direction.

### 3.3. WANS

Materials based on semi-crystalline polymers exhibit a phase separation into crystalline and amorphous regions. These materials are characterized by a complex morphology with multiple, hierarchically organized structural levels that span a wide length scale between several ångströms and hundreds of nanometres (Akpalu, 2010 ▸; Radulescu *et al.*, 2015*b* ▸; Kanaya *et al.*, 2007 ▸). Moreover, the bulk and interlamellar amorphous regions can be functionalized, which results in even more complex morphologies when external stimuli such as humidity, temperature or elongation/com­pression are applied to the sample (Schiavone *et al.*, 2023 ▸). For the characterization of such complex morphologies, a wide length scale has to be covered, which usually requires a combination of different experimental methods in the structural analysis. Simultaneous use of wide- and small-angle scattering methods in the same experiment is necessary for sensitive or expensive samples, if special attention must be paid to sample preparation (composition, quality, amount *etc*.) or *in situ* treatment (temperature, humidity, chemical conditions *etc*.) during the experimental investigation (Tashiro & Sasaki, 2003 ▸). While modern X-ray laboratory diffractometers or beamlines at large-scale facilities can easily and successfully combine ultra-small-angle X-ray scattering (USAXS), SAXS and wide-angle X-ray scattering (WAXS) (Pauw *et al.*, 2021 ▸), in the case of neutron scattering such a combination of methods requires special care. WANS and SANS can be simultaneously performed without technical or organizational difficulties with TOF SANS instruments at spallation or steady-state (reactor) neutron sources. In this case, a broad wavelength band is used and a wide angular range of scattering can be covered by using a large number of detectors, either movable (Heenan *et al.*, 2005 ▸; Zhao *et al.*, 2010 ▸; Sokolova *et al.*, 2019 ▸; Dewhurst *et al.*, 2016 ▸) or placed in fixed positions (Takata *et al.*, 2015 ▸; Koizumi *et al.*, 2020 ▸; Allen, 2023 ▸). On the other hand, equipping a classical pinhole SANS instrument at a research reactor with wide-angle detectors enables the *Q* range to be extended towards higher *Q* values (Heller *et al.*, 2018 ▸) and allows access to the diffraction mode. Thus it is possible to bridge the atomic and mesoscales at the same instrument, provided that the instrument resolution can be tuned to serve the scientific goal.

**Figure 12:**
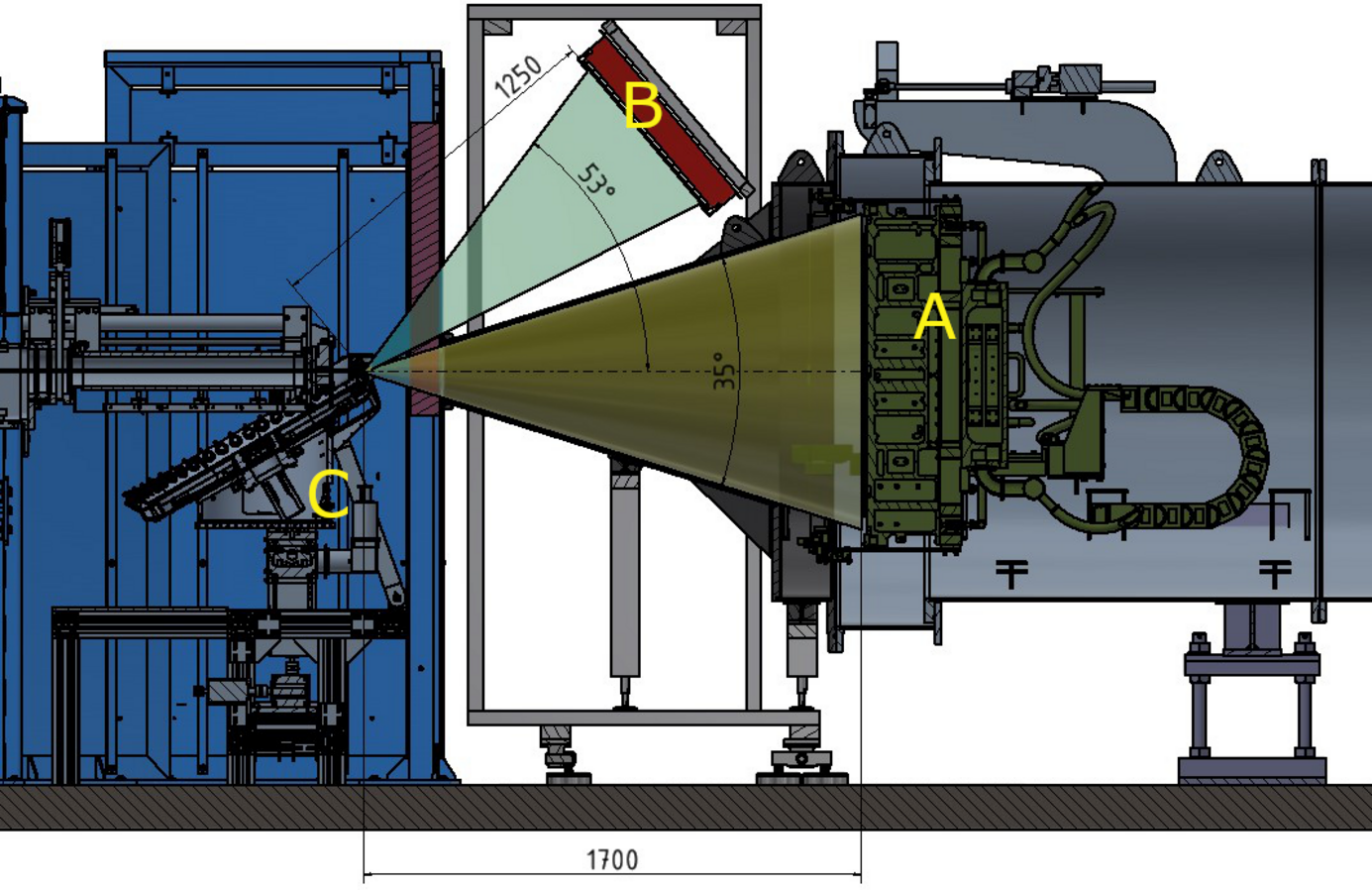
Schematic representation of the SANS and WANS concept at KWS-2: the SANS angular range is shown in dark green (maximum θ_s_ = 17.5°), while the WANS angular range is depicted by the blue–green field (maximum θ_s_ = 53°). The *L*_D_ for the WANS detector and the minimum *L*_D_ for the SANS detector are indicated in mm. The SANS detector at *L*_D_ = 1.7 m is labeled A, the WANS detector at *L*_D_ = 1.25 m is labeled B and the automatic sample changer (carousel with 48 positions) is shown at the sample position (C).

A new detector consisting of an array of ^3^He tubes is currently being tested at JCNS with the aim of installing it for the WANS option at KWS-2. For this purpose, the first 1.7 m-long cylindrical segment of the evacuated tank at KWS-2 is currently being replaced by a conical nose (Fig. S7), which will share the common vacuum with the rest of the tank. This geometry allows the WANS detector to be installed in a fixed inclined position above the transmitted beam axis at a distance *L*_D_ = 1.25 m after the sample (Fig. 12 ▸). The neutrons scattered by the sample can be detected over a wide angular range simultaneously with the SANS detector (up to θ_s_ = 17.5°) and with the WANS detector (up to θ_s_ = 53°), both in continuous and in TOF mode. In this detection geometry, a maximum value in the *Q* range of *Q*_max_ = 2.0 Å^−1^ is achieved, while working with neutrons of wavelength λ = 2.8 Å. A suitable Δ*Q*/*Q* resolution achieved with the resolution chopper (Radulescu *et al.*, 2015*a* ▸) and TOF data acquisition will allow detailed observation of crystalline peaks of semi-crystalline polymer systems, as shown in the recently published proof of principle (Radulescu, 2024 ▸). It is expected that, with this upgrade, measurements of similar quality to that of data collected at the BL-15 Taikan of J-PARC (Schiavone *et al.*, 2023 ▸) will also be possible at KWS-2.

The WANS detector consists of an array of 80 ^3^He tubes and fast readout electronics supplied by Canon Electron Tubes & Devices (CETD, Otawara, Japan) and Clear Pulse (Tokyo, Japan). The ^3^He tubes with a diameter of 6 mm and an active length of 60 cm thus provide a detection field of approximately 50 × 60 cm for the WANS geometry. The tubes are filled with ^3^He and CO_2_ as a stop gas, at a ^3^He partial pressure of 12 atm. According to the manufacturer, the detection efficiency is about 60% for λ = 3 Å and 80% for λ = 7 Å. The WANS detector will operate in air, which makes installation, maintenance and operation very simple. On the basis of experience gained at J-PARC and JRR-3 at the SANS beamlines with the WANS detectors operated in air, a good WANS data quality is expected. However, the concept of the WANS option includes the installation of a helium or argon box in front of the detector to minimize the air path between the sample and the position-sensitive detectors and to reduce the background and beam attenuation due to scattering in air. Use of argon or helium at or near ambient pressure means that the pressure difference between the inside and outside of the box is negligible, so the box can be built with very thin windows. If it proves necessary during the commissioning tests, the helium or argon box can be installed to keep the background at a very low level. To mitigate the effect of the entrance window flange, which creates an angular gap between the SANS and WANS detectors (Fig. 12 ▸), the *Q* overlap between the WANS and SANS data can be adjusted by working with different wavelengths in the range of 2.8–5 Å and performing short measurements with both detectors in the high-flux regime of the instrument, *i.e.* with the SANS detector at *L*_D_ = 1.7 m. As previously reported (Radulescu, 2024 ▸), the instrumental resolution in the *Q* range between 1 and 2 Å^−1^ depends practically only on Δλ/λ, with the collimation contribution having a negligible influence. This opens up the possibility of maximizing the intensity on the sample by using the shorter collimation length *L*_C_ = 2 m, *i.e.* very short measurements, without compromising the quality of the data.

Fig. S8 shows the view of the detector, consisting of the ten 8-packs assembled into a 2D detection array. A second similar ensemble, to be installed symmetrically to the detector shown in Fig. 12 ▸ under the beam axis, may be considered in the near future. The data acquisition (DAQ) middleware was developed at JCNS to communicate with the CETD firmware installed on the detector modules and to enable the correct data acquisition and sorting conditions to be set. Successful tests of a prototype of an 8-pack of ^3^He tubes were performed at JCNS and FRM II in 2023, motivating the purchase and installation of a full system at KWS-2. It should also be mentioned at this point that the ^3^He tube detectors supplied by CETD at the SANS-J instrument at the JRR-3 reactor in Japan (Noda *et al.*, 2016 ▸; Kumada *et al.*, 2023 ▸) and at the BL-15 Taikan at J-PARC (Takata *et al.*, 2015 ▸) are successfully operating in both SANS and WANS measurement geometries, which strongly supported the choice of this type of detector for the WANS option at KWS-2. The test of the prototype in 2023 and of the complete system in 2024 was performed with a ^252^Cf neutron source at the FRM II in Garching.

**Figure 13:**
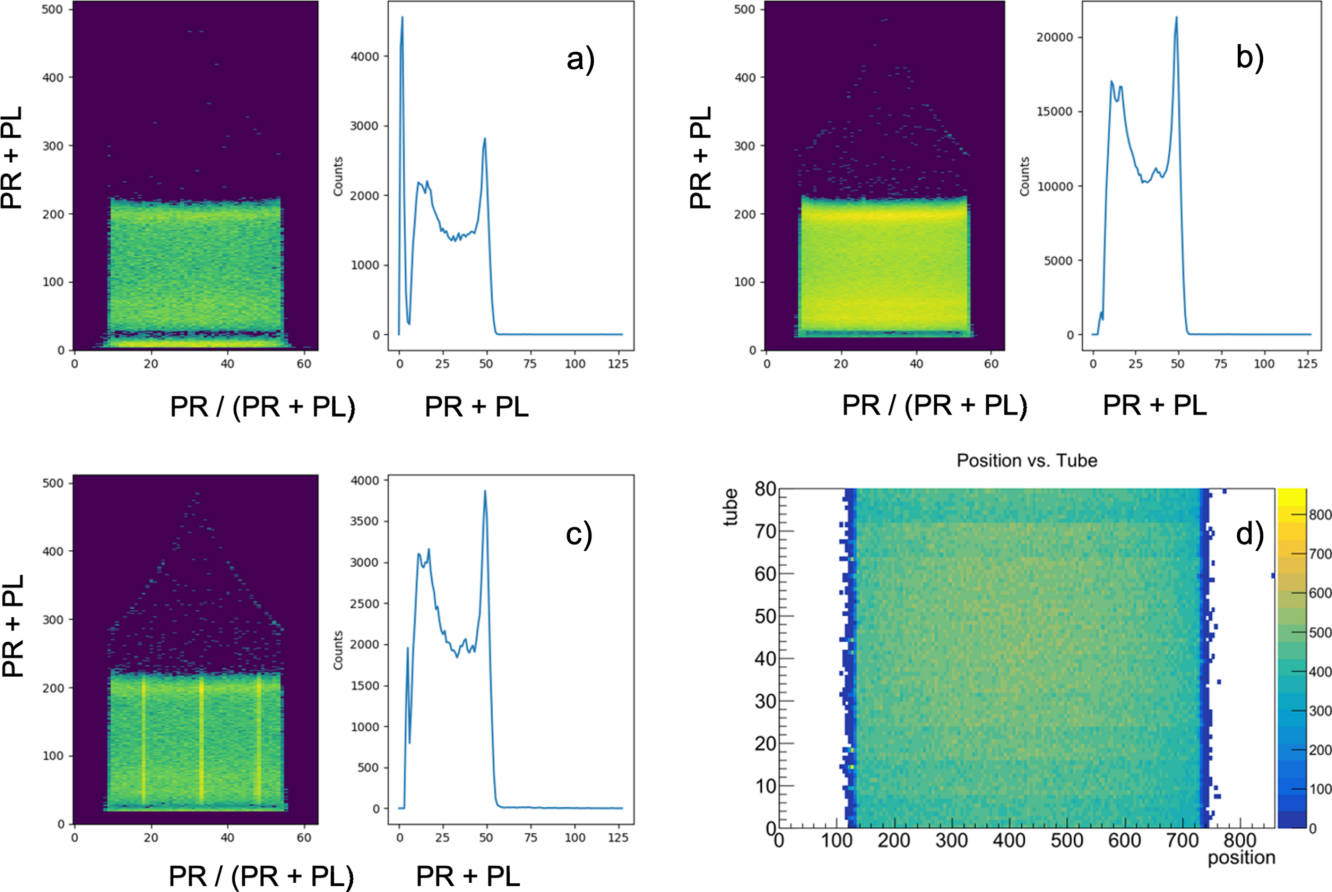
Examples of position versus amplitude spectra [colored panels in (*a*), (*b*) and (*c*)] and pulse height spectra [blue curves in (*a*), (*b*) and (*c*)] measured with the detector during the test with the ^252^Cf neutron source: (*a*) full spectrum; (*b*) the spectrum with the γ peak at lower channels (< 10) sorted out; (*c*) results with the absorbing mask with three narrow slits (3 mm) in front of the detector, with the slits transverse to the horizontal ^3^He tubes. In (*c*), partial γ-peak discrimination was used during the middleware DAQ test. The flooding test of the entire detector with the ^252^Cf neutron source is shown in (*d*). Position was obtained from the ratio of the right amplitude (PR) to the total pulse height (PR + PL).

**Figure 14:**
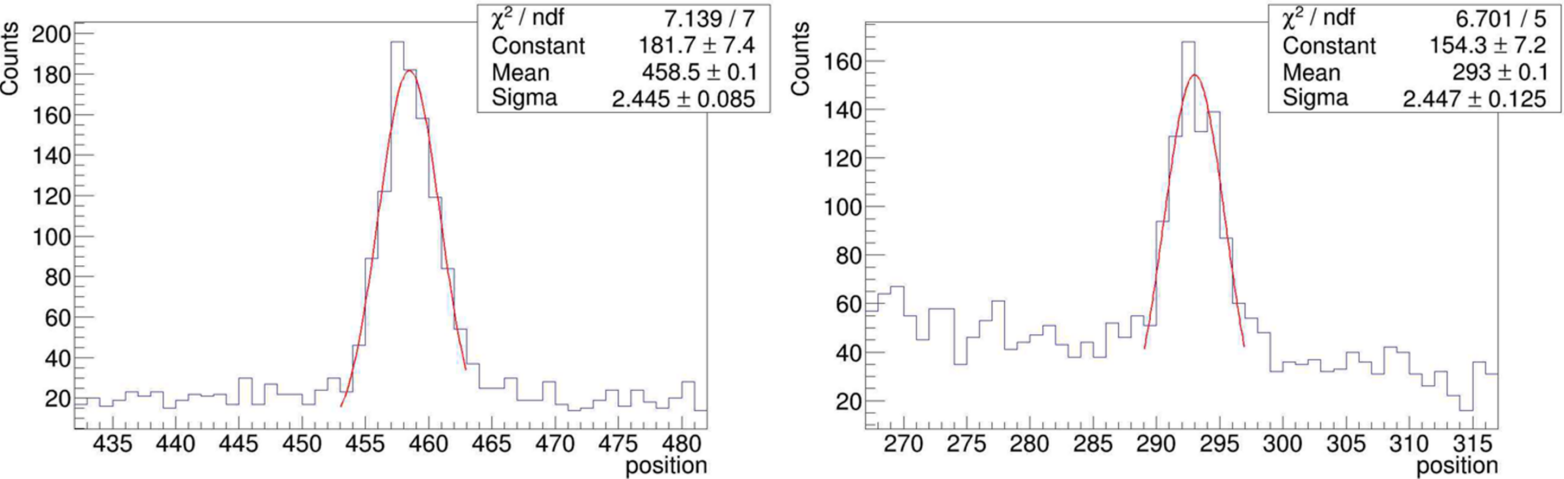
Gaussian fit of the peaks detected during irradiation with neutrons from the ^252^Cf source along a ^3^He tube covered with an absorbing mask with local slits (2 mm width) across the tube: the left peak corresponds to the slit in the center of the tube, while the right peak originates from the slit at the left end of the tube. FWHM = 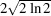 Sigma is an indicator of the position resolution of the ^3^He tube along the tube axis.

The usual tests as described in our previous work on the optimization, installation and commissioning of the SANS main detector (Radulescu *et al.*, 2016 ▸; Houston *et al.*, 2018 ▸) were also performed with the CETD detectors. Figs. 13 ▸(*a*)–13 ▸(*c*) show examples of the typical position/amplitude results and pulse height spectra collected in different setting conditions of the detector, and an image of detector flooding with the ^252^Cf neutron source is shown in Fig. 13 ▸(*d*). The typical pulse height distribution spectrum collected during the tests without γ discrimination is shown in Fig. 13 ▸(*a*), right panel. The full energy peak can be easily observed just above channel No. 50, while the proton and triton peaks are yielded at lower channels. The narrow and intense γ peak when not discriminated is observable at very low channels (<10), while it is sorted out [Fig. 13 ▸(*b*), right panel] when the γ discrimination through the JCNS DAQ middleware is applied. Full detector illumination data [Fig. 13 ▸(*d*)] were interpreted in terms of measured events across position and amplitude (energy) as exemplified in Fig. S9, which confirmed the good quality of the position response of the detector. The position resolution was investigated on the prototyped module by using a Cd mask with three slits carved across the 8-pack for a slit width of 3 mm (Fig. S10), while for the full detector a boron carbide mask with three slits of 2 mm width was used. Fig. 14 ▸ displays an example of a Gaussian fit to peaks measured at two positions along a tube in the full detector, which yielded a position resolution of about FWHM ≃ 2.355σ = 5.75 mm in the middle and towards the ends of the tube. However, this may be considered as over-evaluated due to the compact measurement geometry with the detector placed too close to the ^252^Cf source. Thus the very wide divergence of the beam falling onto the detector and the relatively large width of the slits used for the experiment lead to an overestimated position resolution. The precise position resolution of the detector along the ^3^He tubes remains to be confirmed again with neutrons at KWS-2 where the high intensity will enable the use of a much narrower slit opening (1 mm width). Following the laboratory tests, it can be considered that the newly implemented detector perfoms well. The installation at KWS-2 is currently in progress, with the goal of having the WANS option operational for the expected restart of the FRM II reactor at the end of 2025.

## 4. Conclusions

New upgrades recently completed or in the process of being installed at the JCNS KWS-2 SANS diffractometer at MLZ are reported. These are specific upgrades to the sample environment and detection capabilities to enhance the performance of the instrument for selected scientific purposes in biology and energy applications. An *in situ* SEC complementarity has been optimized for the specific configuration at KWS-2 with the FPLC outside the sample area, to provide the instrument with biological samples of similar quality to other SAXS and SANS beamlines with SEC purification in the beam. For controlled in-beam hydration of solid ionomers or biological membranes, a versatile dew point generator dedicated to neutron scattering was calibrated and commissioned with Nafion membranes of known ionic conductivity measured with a variable RH and T conductivity cell. To bridge the atomic and mesoscale at KWS-2, a WANS option with a ^3^He tube detector is under construction, for which a quality assessment with a ^252^Cf neutron source was carried out at JCNS and FRM II. With a *Q*_max_ = 2.0 Å^−1^ and an adjustable resolution provided by the instrument’s chopper in TOF mode, semi-crystalline materials or small biological morphologies can be structurally characterized in a very thorough way at the same beamline. All these innovations at KWS-2 will be fully operational when the FRM II reactor is brought back into operation, which is planned for the end of 2025. The planned restart of the reactor with only thermal neutrons in the first phase due to the delayed production of the new cold source will not have a severe impact on the new upgrades at KWS-2, although a lower operating flux than in the standard configuration is expected. A combined strategy is planned at KWS-2 to overcome the flux reduction caused by this temporary reactor configuration: (i) an increased use of shorter wavelengths around 3 Å, less affected by the absence of a cold source, while using a relaxed resolution with a dedicated velocity selector for Δλ/λ ≥ 20%; (ii) the repetition of measurements in the mid and high *Q* range to obtain good data statistics, which is a typical approach for SEC–SANS experiments; (iii) the use of neutron lenses and large sample sizes (up to 5 cm diameter), easily achievable for gel, colloid, micellar or membrane samples, which allows the intensity on the sample to be increased by a factor of up to 10 while maintaining the resolution as for pinhole geometry, thus compensating for the flux decrease in low-*Q* measurements.

## Supplementary Material

Supporting information. DOI: 10.1107/S160057672500158X/ge5168sup1.pdf

## Data Availability

The data supporting the results reported in this article can be accessed upon request from the corresponding author.

## References

[bb2] Adamski, M., Peressin, N. & Holdcroft, S. (2021). *Mater. Adv.* **2**, 4966–5005.

[bb3] Akpalu, Y. V. (2010). *Polym. Rev.* **50**, 1–13.

[bb4] Allahyarov, E. & Taylor, P. L. (2009). *J. Phys. Chem. B*, **113**, 610–617. 10.1021/jp8047746 19115809

[bb5] Allen, A. J. (2023). *J. Appl. Cryst.* **56**, 787–800. 10.1107/S1600576723003898 PMC10241057 37284276

[bb6] Amann, M., Willner, L., Stellbrink, J., Radulescu, A. & Richter, D. (2015). *Soft Matter*, **11**, 4208–4217. 10.1039/c5sm00469a 25892401

[bb7] Arima-Osonoi, H., Takata, S., Kasai, S., Ohuchi, K., Morikawa, T., Miyata, N., Miyazaki, T., Aoki, H., Iwase, H., Hiroi, K., Ogura, M., Kikuchi, T., Takashina, H. & Sakayori, T. (2023). *J. Appl. Cryst.* **56**, 1802–1812.

[bb8] Balacescu, L., Brandl, G. & Radulescu, A. (2021). *J. Appl. Cryst.* **54**, 1217–1224. 10.1107/S1600576721006610 PMC8366428 34429724

[bb9] Biehl, R. (2019). *PLoS One*, **14**, e0218789. 10.1371/journal.pone.0218789 PMC6590829 31233549

[bb10] Bose, S., Kuila, T., Nguyen, T. X. H., Kim, N. H., Lau, K. & Lee, J. H. (2011). *Prog. Polym. Sci.* **36**, 813–843.

[bb11] Bucciarelli, S., Midtgaard, S. R., Nors Pedersen, M., Skou, S., Arleth, L. & Vestergaard, B. (2018). *J. Appl. Cryst.* **51**, 1623–1632. 10.1107/S1600576718014462 PMC6276278 30546289

[bb12] Dewhurst, C. D., Grillo, I., Honecker, D., Bonnaud, M., Jacques, M., Amrouni, C., Perillo-Marcone, A., Manzin, G. & Cubitt, R. (2016). *J. Appl. Cryst.* **49**, 1–14.

[bb13] Graewert, M. A., Da Vela, S., Gräwert, T. W., Molodenskiy, D. S., Blanchet, C. E., Svergun, D. I. & Jeffries, C. M. (2020). *Crystals*, **10**, 975.

[bb14] Grudinin, S., Garkavenko, M. & Kazennov, A. (2017). *Acta Cryst.* D**73**, 449–464. 10.1107/S2059798317005745 28471369

[bb15] Grzybek, P., Dudek, G. & van der Bruggen, B. (2024). *Chem. Eng. J.* **495**, 153500.

[bb17] Heenan, R. K., King, S. M., Turner, D. S. & Treadgold, J. R. (2005). *Proc. ICANS*, XVII, 780–785.

[bb18] Heller, W. T., Cuneo, M., Debeer-Schmitt, L., Do, C., He, L., Heroux, L., Littrell, K., Pingali, S. V., Qian, S., Stanley, C., Urban, V. S., Wu, B. & Bras, W. (2018). *J. Appl. Cryst.* **51**, 242–248.

[bb19] Houston, J. E., Brandl, G., Drochner, M., Kemmerling, G., Engels, R., Papagiannopoulos, A., Sarter, M., Stadler, A. & Radulescu, A. (2018). *J. Appl. Cryst.* **51**, 323–336. 10.1107/S1600576718004132 PMC5884387 29657566

[bb20] Inoue, R., Nakagawa, T., Morishima, K., Sato, N., Okuda, A., Urade, R., Yogo, R., Yanaka, S., Yagi-Utsumi, M., Kato, K., Omoto, K., Ito, K. & Sugiyama, M. (2019). *Sci. Rep.* **9**, 12610. 10.1038/s41598-019-48911-w PMC6717197 31471544

[bb21] Johansen, N. T., Pedersen, M. C., Porcar, L., Martel, A. & Arleth, L. (2018). *Acta Cryst.* D**74**, 1178–1191. 10.1107/S2059798318007180 30605132

[bb22] Jordan, A., Jacques, M., Merrick, C., Devos, J., Forsyth, V. T., Porcar, L. & Martel, A. (2016). *J. Appl. Cryst.* **49**, 2015–2020. 10.1107/S1600576716016514 PMC5139991 27980509

[bb23] Kanaya, T., Matsuba, G., Ogino, Y., Nishida, K., Shimizu, H. M., Shinohara, T., Oku, T., Suzuki, J. & Otomo, T. (2007). *Macromolecules*, **40**, 3650–3654.

[bb24] Kaneko, F., Schiavone, M. M., Iwase, H., Takata, S., Allgaier, J. & Radulescu, A. (2024). *Polymer*, **295**, 126771.

[bb25] Klein, M., Perrin, J.-C., Leclerc, S., Guendouz, L., Dillet, J. & Lottin, O. (2013). *Diffusion Fundamentals*, **18**, 1–4.

[bb26] Koizumi, S., Noda, Y., Maeda, T., Inada, T., Ueda, S., Fujisawa, T., Izunome, H., Robinson, R. A. & Frielinghaus, H. (2020). *QuBS*, **4**, 42.

[bb27] Krugmann, B., Radulescu, A., Appavou, M.-S., Koutsioubas, A., Stingaciu, L. R., Dulle, M., Förster, S. & Stadler, A. (2020). *Sci. Rep.* **10**, 16691. 10.1038/s41598-020-73671-3 PMC7542173 33028889

[bb28] Kumada, T., Motokawa, R., Oba, Y., Nakagawa, H., Sekine, Y., Micheau, C., Ueda, Y., Sugita, T., Birumachi, A., Sasaki, M., Hiroi, K. & Iwase, H. (2023). *J. Appl. Cryst.* **56**, 1776–1783.

[bb70] Le Trong, I., Freitag, S., Klumb, L. A., Chu, V., Stayton, P. S. & Stenkamp, R. E. (2003). *Acta Cryst.* D**59**, 1567–1573. 10.1107/s0907444903014562 12925786

[bb29] Luo, X., Rojas-Carbonell, S., Yan, Y. & Kusoglu, A. (2020). *J. Membr. Sci.* **598**, 117680.

[bb30] Martel, A., Cocho, C., Caporaletti, F., Jacques, M., El Aazzouzi, A., Lapeyre, F. & Porcar, L. (2023). *J. Appl. Cryst.* **56**, 994–1001. 10.1107/S1600576723004119 PMC10405598 37555207

[bb31] Mendil-Jakani, H., Pouget, S., Gebel, G. & Pintauro, P. N. (2015). *Polymer*, **63**, 99–107.

[bb34] Noda, Y., Koizumi, S. & Yamaguchi, D. (2016). *J. Appl. Cryst.* **49**, 128–138. 10.1107/S1600576716016472 PMC5139992 27980510

[bb35] Pabst, F., Kraus, J., Kloth, S., Steinrücken, E., Kruteva, M., Radulescu, A., Vogel, M. & Blochowicz, T. (2021). *J. Chem. Phys.* **155**, 174501. 10.1063/5.0066180 34742203

[bb36] Pace, C. N., Vajdos, F., Fee, L., Grimsley, G. & Gray, T. (1995). *Protein Sci.* **4**, 2411–2423. 10.1002/pro.5560041120 PMC2143013 8563639

[bb37] Park, J. K., Li, J., Divoux, G. M., Madsen, L. A. & Moore, R. (2011). *Macromolecules*, **44**, 5701–5710.

[bb38] Pauw, B. R., Smith, A. J., Snow, T., Shebanova, O., Sutter, J. P., Ilavsky, J., Hermida-Merino, D., Smales, G. J., Terrill, N. J., Thünemann, A. F. & Bras, W. (2021). *J. Synchrotron Rad.* **28**, 824–833. 10.1107/S1600577521003313 PMC8127376 33949990

[bb39] Pedersen, J. S., Posselt, D. & Mortensen, K. (1990). *J. Appl. Cryst.* **23**, 321–333.

[bb40] Puig-Rigall, J., Blanco-Prieto, M. J., Radulescu, A., Dreiss, C. A. & González-Gaitano, G. (2021). *J. Colloid Interface Sci.* **582**, 353–363. 10.1016/j.jcis.2020.08.004 32858401

[bb41] Puig-Rigall, J., Fernández-Rubio, C., González-Benito, J., Houston, J. E., Radulescu, A., Nguewa, P. & González-Gaitano, G. (2020). *Int. J. Pharm.* **578**, 119057. 10.1016/j.ijpharm.2020.119057 31991188

[bb42] Radulescu, A. (2024). *J. Appl. Cryst.* **57**, 1040–1046. 10.1107/S160057672400493X PMC11299620 39108807

[bb43] Radulescu, A., Arend, N., Drochner, M., Ioffe, A., Kemmerling, G., Ossovyi, V., Staringer, S., Vehres, G., McKinny, K., Olechnowicz, B. & Yen, D. (2016). *J. Phys. Conf. Ser.* **746**, 012026.

[bb44] Radulescu, A., Goerigk, G., Fetters, L. & Richter, D. (2015*b*). *J. Appl. Cryst.* **48**, 1860–1869. 10.1107/S1600576715019226 PMC4665662 26664344

[bb45] Radulescu, A., Pipich, V., Frielinghaus, H. & Appavou, M. S. (2012*a*). *J. Phys. Conf. Ser.* **351**, 012026.

[bb46] Radulescu, A., Pipich, V. & Ioffe, A. (2012*b*). *Nucl. Instrum. Methods Phys. Res. A*, **689**, 1–6.

[bb47] Radulescu, A., Székely, N. K., Polachowski, S., Leyendecker, M., Amann, M., Buitenhuis, J., Drochner, M., Engels, R., Hanslik, R., Kemmerling, G., Lindner, P., Papagiannopoulos, A., Pipich, V., Willner, L., Frielinghaus, H. & Richter, D. (2015*a*). *J. Appl. Cryst.* **48**, 1849–1859. 10.1107/S1600576715019019 PMC4665661 26664343

[bb71] Raj, S. B., Ramaswamy, S. & Plapp, B. V. (2014). *Biochemistry*, **53**, 5791–5803. 10.1021/bi5006442 PMC4165444 25157460

[bb48] Rubatat, L. & Diat, O. (2007). *Macromolecules*, **40**, 9455–9462.

[bb49] Ryan, T. M., Trewhella, J., Murphy, J. M., Keown, J. R., Casey, L., Pearce, F. G., Goldstone, D. C., Chen, K., Luo, Z., Kobe, B., McDevitt, C. A., Watkin, S. A., Hawley, A. M., Mudie, S. T., Samardzic Boban, V. & Kirby, N. (2018). *J. Appl. Cryst.* **51**, 97–111.

[bb50] Schiavone, M.-M., Lamparelli, D. H., Daniel, C., Golla, M., Zhao, Y., Iwase, H., Arima-Osonoi, H., Takata, S., Szentmiklosi, L., Maroti, B., Allgaier, J. & Radulescu, A. (2023). *J. Appl. Cryst.* **56**, 947–960. 10.1107/S1600576723005496 PMC10405591 37555213

[bb51] Shih, O., Liao, K.-F., Yeh, Y.-Q., Su, C.-J., Wang, C.-A., Chang, J.-W., Wu, W.-R., Liang, C.-C., Lin, C.-Y., Lee, T.-H., Chang, C.-H., Chiang, L.-C., Chang, C.-F., Liu, D.-G., Lee, M.-H., Liu, C.-Y., Hsu, T.-W., Mansel, B., Ho, M.-C., Shu, C.-Y., Lee, F., Yen, E., Lin, T.-C. & Jeng, U. (2022). *J. Appl. Cryst.* **55**, 340–352. 10.1107/S1600576722001923 PMC8985603 35497659

[bb52] Sokolova, A., Whitten, A. E., de Campo, L., Christoforidis, J., Eltobaji, A., Barnes, J., Darmann, F. & Berry, A. (2019). *J. Appl. Cryst.* **52**, 1–12.

[bb53] Sone, Y., Ekdunge, P. & Simonsson, D. (1996). *J. Electrochem. Soc.* **143**, 1254–1259.

[bb54] Song, H., Liu, Y., Zhang, W., Zhang, X., Yin, X., Li, J. & Wu, G. (2022). *J. Power Sources*, **539**, 231623.

[bb55] Takata, S., Suzuki, J., Shinohara, T., Oku, T., Tominaga, T., Ohishi, K., Iwase, H., Nakatani, T., Inamura, Y., Ito, T., Suzuya, K., Aizawa, K., Arai, M., Otomo, T. & Sugiyama, M. (2015). *JPS Conf. Proc.* **8**, 036020.

[bb56] Talley, S. J., Vivod, S. L., Nguyen, G. A., Meador, M. A. B., Radulescu, A. & Moore, R. B. (2019). *Appl. Mater. Interfaces*, **11**, 31508–31519. 10.1021/acsami.9b09699 31379150

[bb57] Tashiro, K. & Sasaki, S. (2003). *Polymer*, **28**, 451–519.

[bb58] Thureau, A., Roblin, P. & Pérez, J. (2021). *J. Appl. Cryst.* **54**, 1698–1710.

[bb59] Wang, H., Holmberg, B. A., Huang, L., Wang, Z., Mitra, A., Norbeck, J. M. & Yan, Y. (2002). *J. Mater. Chem.* **12**, 834–837.

[bb62] Yoshimura, K., Hiroki, A., Radulescu, A., Noda, Y., Koizumi, S., Zhao, Y. & Maekawa, Y. (2024). *Macromolecules*, **57**, 1998–2007.

[bb63] Zhao, J. K., Gao, C. Y. & Liu, D. (2010). *J. Appl. Cryst.* **43**, 1068–1077.

[bb64] Zhao, Y., Yoshimura, K., Motegi, T., Hiroki, A., Radulescu, A. & Maekawa, Y. (2021). *Macromolecules*, **54**, 4128–4135.

[bb65] Zhao, Y., Yoshimura, K., Sawada, S., Motegi, T., Hiroki, A., Radulescu, A. & Maekawa, Y. (2022). *Macromolecules*, **55**, 7100–7109.

