# Supplementary material for: Upgrade of the KWS-2 high-intensity/extended-Q-range SANS diffractometer of JCNS for soft matter and biophysics: in situ SEC, controlled in situ RH/T variation and WANS detection
Source: J Appl Crystallogr. 2025 Mar 19;58(Pt 2):581–94. doi: 10.1107/S160057672500158X (PMC11957400; doi:10.1107/S160057672500158X)
Supplement: Supplementary file 1 [file j-58-00581-sup1.pdf]

JOURNAL OF APPLIED CRYSTALLOGRAPHY

Volume 58 (2025)

Supporting information for article:

Upgrade of the KWS-2 High-Intensity / Extended Q-range SANS Diffractometer of JCNS for Soft-Matter and Biophysics: *in-situ* SEC, controlled *in-situ* RH/T variation and WANS detection

Jia-Jhen Kang, Ralf Biehl, Georg Brandl, Helmut Korb, Kimio Yoshimura, Vladimir Ossovyi, Andreas Nebel, Jacqueline Lippertz, Ralf Engels, Gunter Kemmerling, Alexander Zaft, Hiroki Iwase, Hiroshi Arima-Osonoi, Shin-ichi Takata, Alexander Weber, Simon Staringer, Baohu Wu, Yue Zhao, Stefan Mattauch and Aurel Radulescu

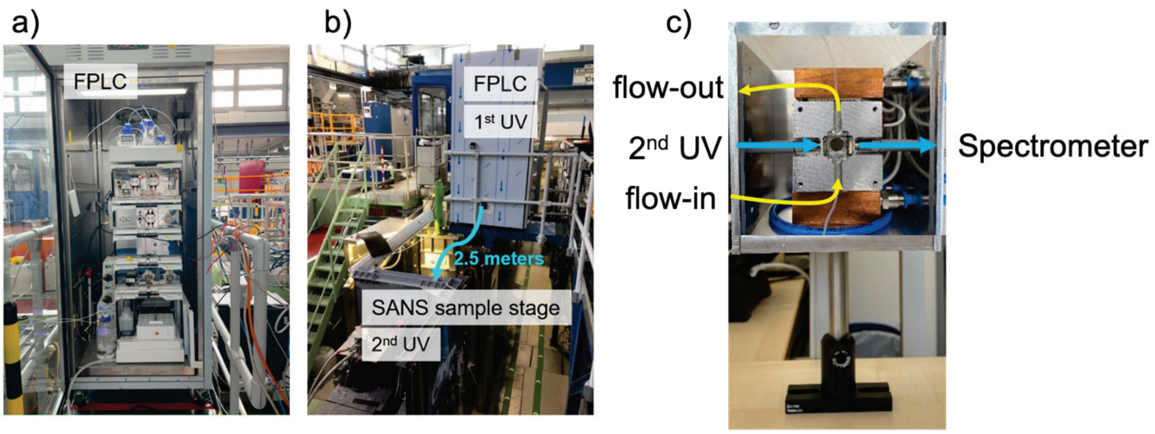

Figure S1 Pictures of different parts of the SEC-SANS setup at the KWS-2 instrument at MLZ. (a) The FPLC instrument in a fridge. (b) The spatial arrangement: the FPLC instrument is located at an elevated platform above the SANS sample stage. (c) The SANS cuvette holder that comes on the sample stage: the yellow arrows indicate the sample flowing through the customized quartz cuvette while the blue arrows shows the UV beam direction. The temperature on the cell can be varied in a controlled way by using the Peltier elements mounted between the Al-holder and the copper oven (with water cooling).

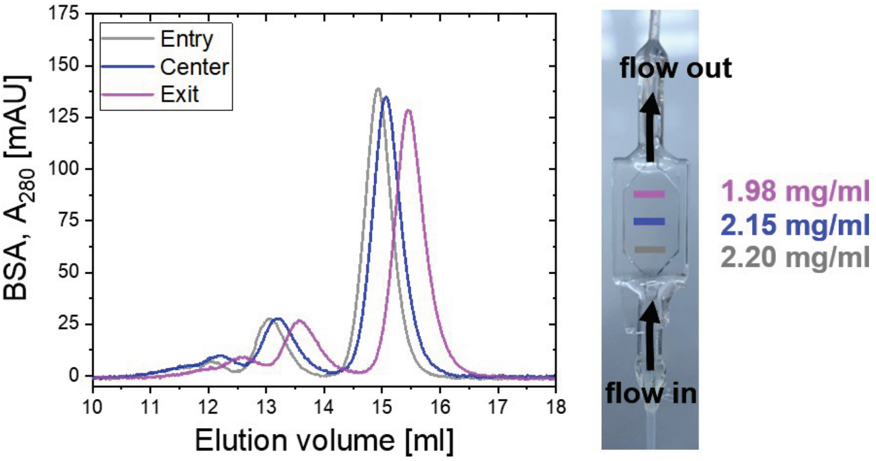

Figure S2 Elution profiles of 10 mg/ml bovine serum albumin, BSA, when the 2nd UV detects at the front window of the flow cell at different positions as indicated, with the corresponding protein concentration written aside.

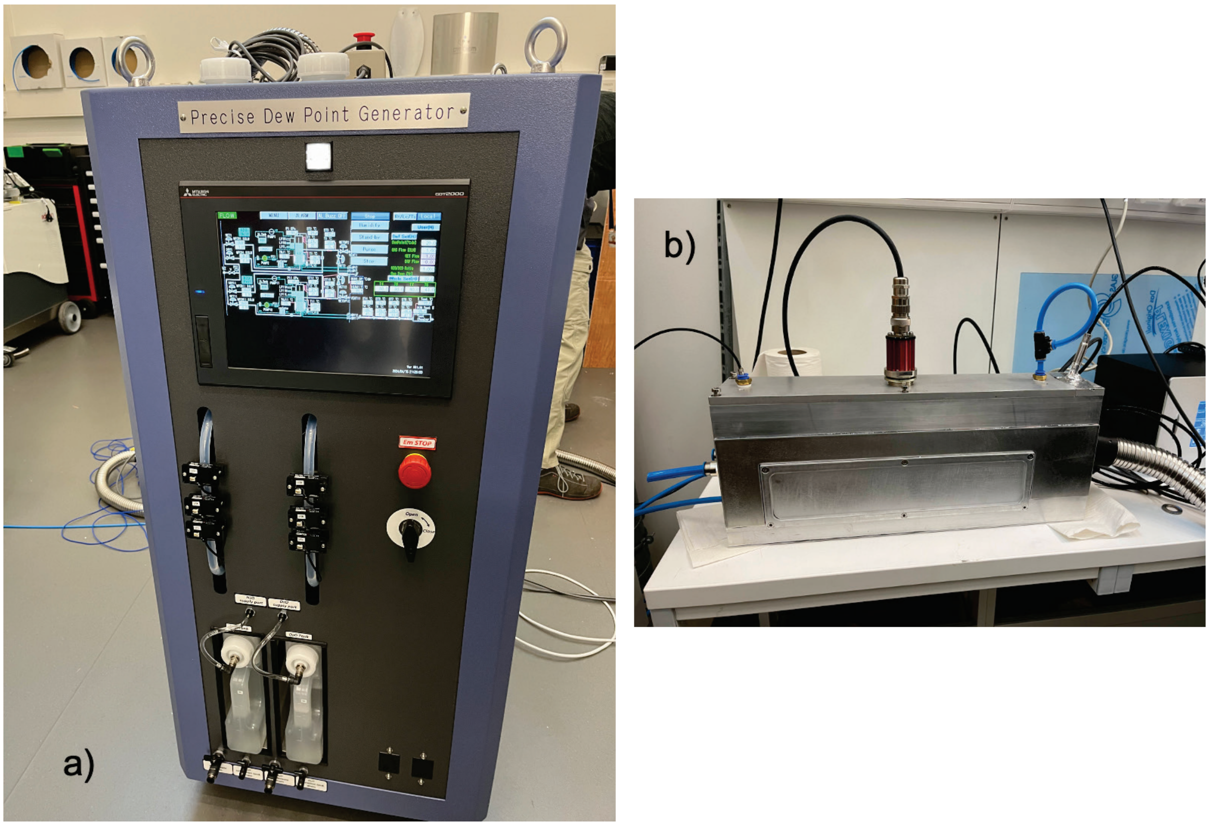

Figure S3 The precise dew point generator with two reservoirs for in-situ changing the contrast in the sample by providing vapor of either H2O, D2O or any H2O/D2O combination (left) and the multiposition sample chamber that works together with the generator. The dew point mirror sensor setup is visible in top of the chamber. Photos are taken during the calibration test carried out at JCNS.

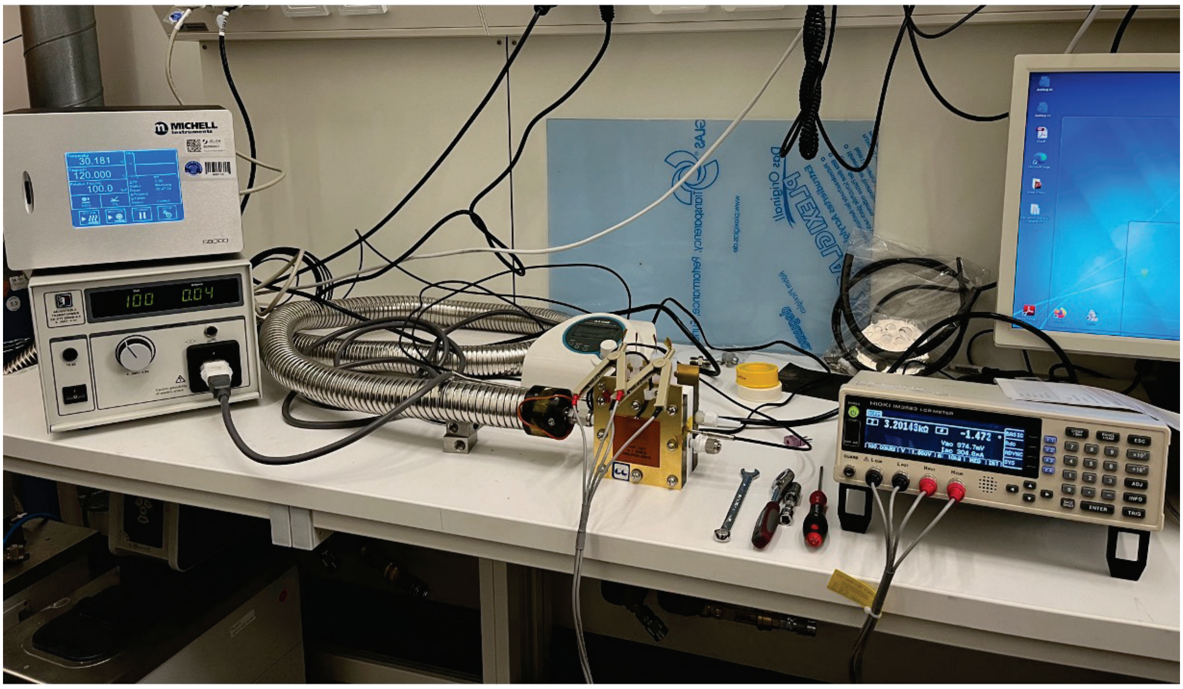

Figure S4 Experimental arrangement at JCNS for measuring the conductivity on the Nafion membranes in variable RH and T conditions. The conductivity cell (Electrochem Inc.) is the yellow device and it is fed with vapors from the dew point generator through the silver tubing coming from the left side. The temperature on the cell is varied by using the device on back side of the cell with the controller placed on the far left side. The LCR meter is the device on the far right side.

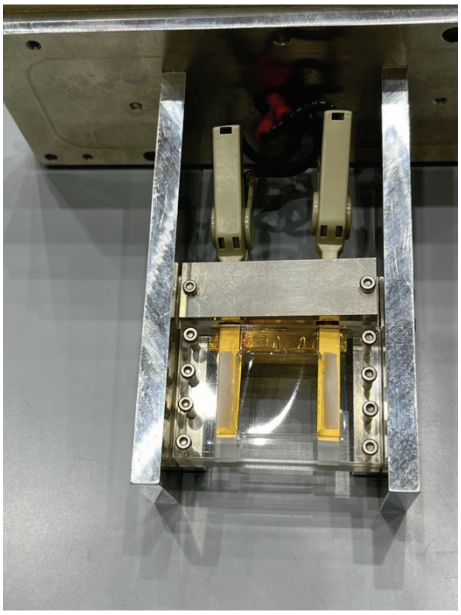

Figure S5 The humidity/conductivity cell (inner elements) of BL-15 Taikan, J-PARC, for simultaneous use with SANS with an as-received Nafion film installed between the electrodes to be exposed to controlled relative humidity and temperature using a single-position humidity chamber.

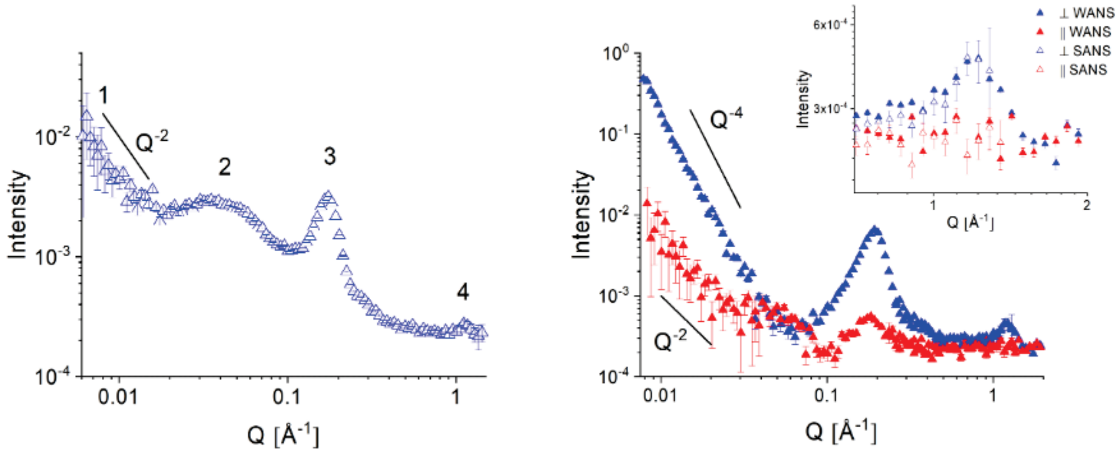

Figure S6 SANS patterns from as received (left) and uni-axially deformed (right) Nafion membranes collected at 80 °C and RH = 85%. Discussion of the results can be found in the main text. The detailed work of simultaneous SANS and in-situ conductivity measurements on polymer electrolyte mebranes at different RH and T conditions will be reported in a forthcoming publication.

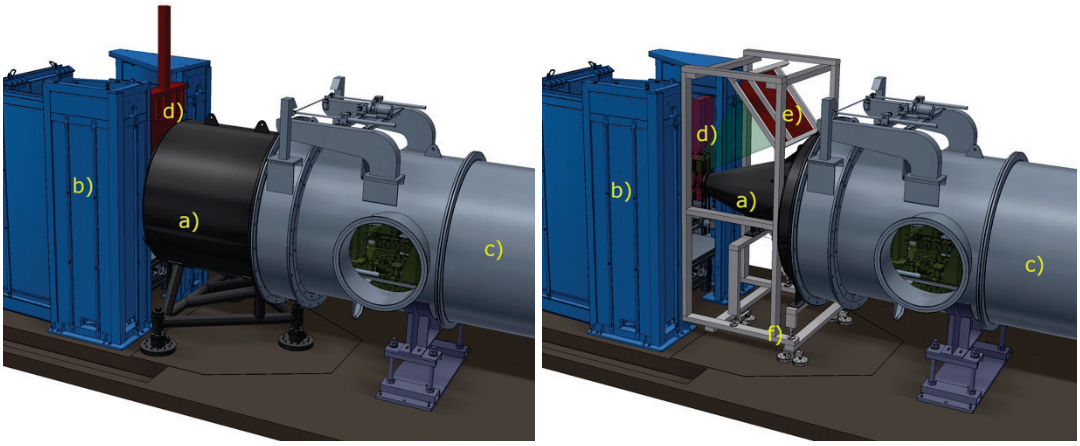

Figure S7 Schematic representation of the KWS-2 components at the sample position and the detection elements at L_D = 1.5-2 m after the sample. Left side - the old setup with a) the cylindrical first segment of the evacuated tank, b) the lead shield around the sample position, c) the rest of the evacuated tank and d) the vacuum shutter behind the entrance window. Right side - the current under construction setup: the new elements are d) the mechanical protection in front of the entrance window, which moves vertically in a controlled fashion and is interlocked with the access door to the sample position to protect the window from mechanical shocks when work is performed at the sample position, e) the WANS detection panel and f) the support frame of the WANS detection panel, which can be equipped with additional shielding elements.

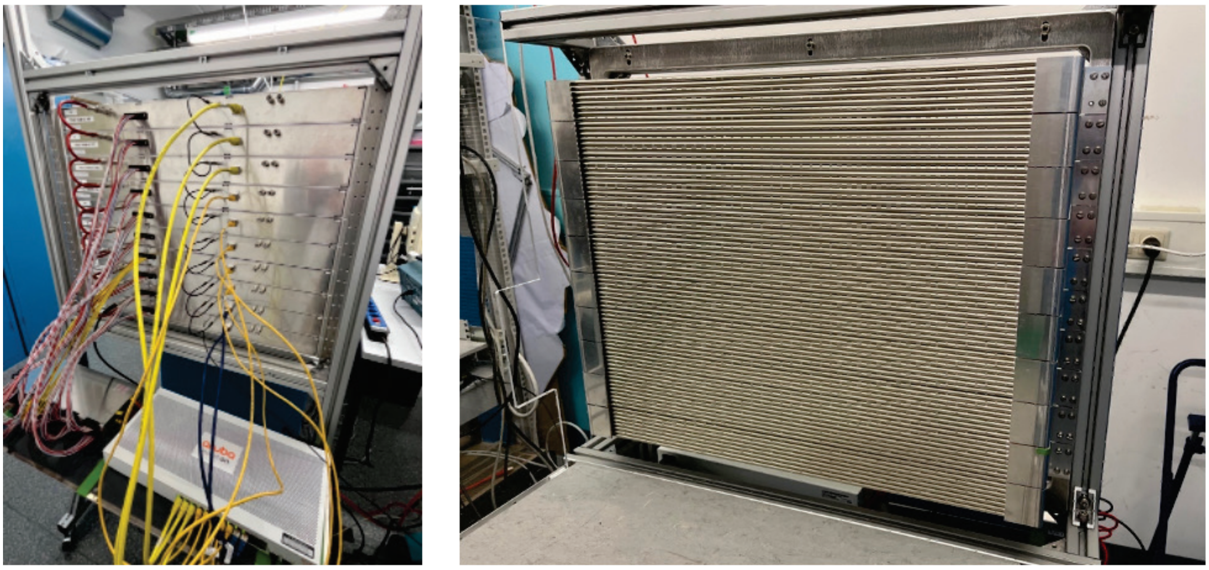

Figure S8 The ^{3}He detector tubes array during the test with the ^{252}Cf source at FRM II, MLZ Garching: the backside of the detector with the signal and power cables (left) and the front side (right).

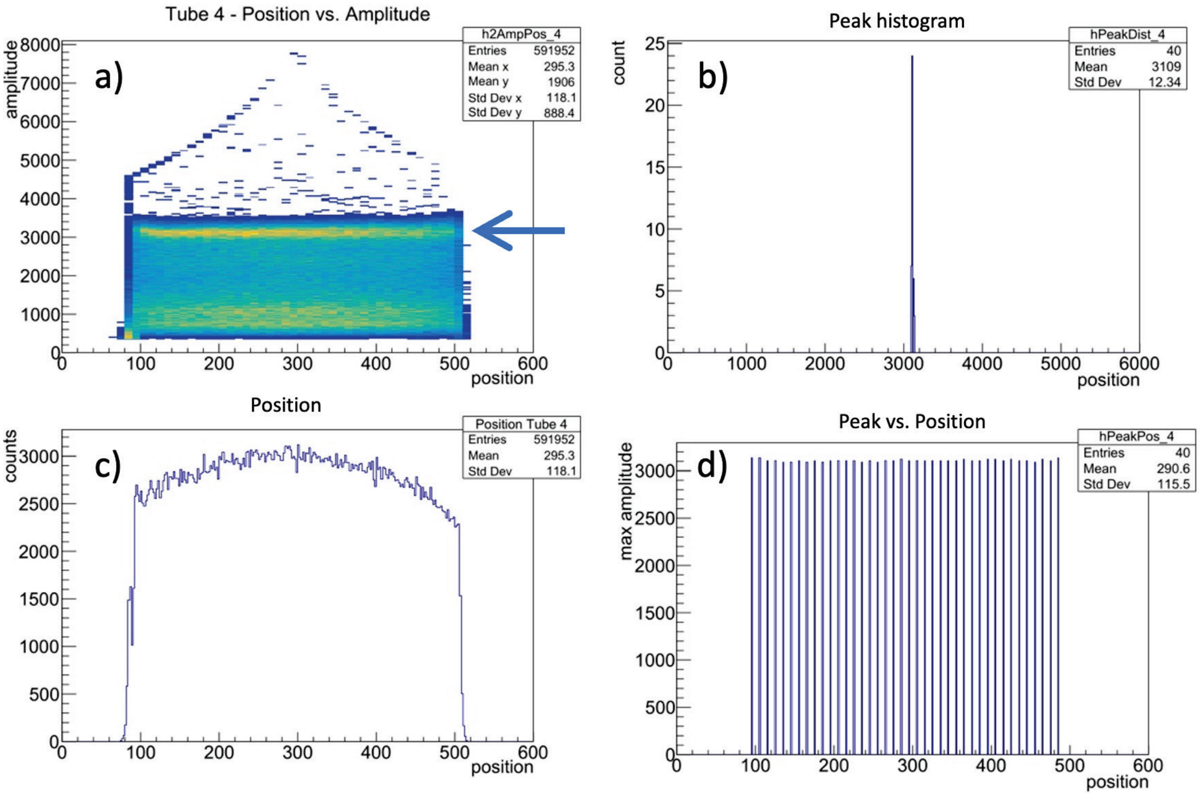

Figure S9 Example of analysis of measured data with the detector: a) position vs. amplitude; b) neutron peak distribution (indicated by the arrow in a)); c) integrated intensity vs. position; d) neutron peak intensity vs. position. For all ^{3}He tubes the neutron peak distribution was less than 5%.

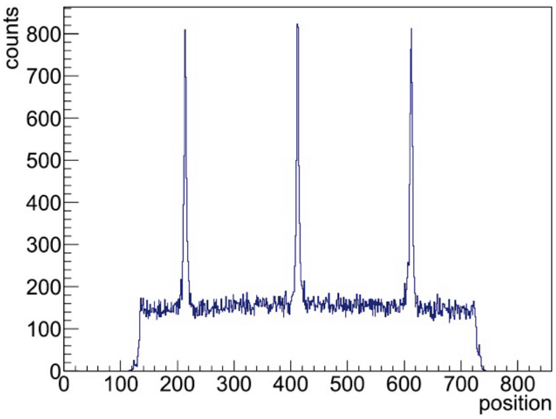

Figure S10 Example of data measured on detector using the Cd-mask with three narrow slits (3 mm); the peaks may be used for the estimation of the detector resolution at different positions along the ^{3}He tube.
